# Homeobox NKX2-3 promotes marginal-zone lymphomagenesis by activating B-cell receptor signalling and shaping lymphocyte dynamics

**DOI:** 10.1038/ncomms11889

**Published:** 2016-06-14

**Authors:** Eloy F. Robles, Maria Mena-Varas, Laura Barrio, Sara V. Merino-Cortes, Péter Balogh, Ming-Qing Du, Takashi Akasaka, Anton Parker, Sergio Roa, Carlos Panizo, Idoia Martin-Guerrero, Reiner Siebert, Victor Segura, Xabier Agirre, Laura Macri-Pellizeri, Beatriz Aldaz, Amaia Vilas-Zornoza, Shaowei Zhang, Sarah Moody, Maria Jose Calasanz, Thomas Tousseyn, Cyril Broccardo, Pierre Brousset, Elena Campos-Sanchez, Cesar Cobaleda, Isidro Sanchez-Garcia, Jose Luis Fernandez-Luna, Ricardo Garcia-Muñoz, Esther Pena, Beatriz Bellosillo, Antonio Salar, Maria Joao Baptista, Jesús Maria Hernandez-Rivas, Marcos Gonzalez, Maria Jose Terol, Joan Climent, Antonio Ferrandez, Xavier Sagaert, Ari M. Melnick, Felipe Prosper, David G. Oscier, Yolanda R. Carrasco, Martin J. S. Dyer, Jose A. Martinez-Climent

**Affiliations:** 1 Division of Hemato-Oncology, Center for Applied Medical Research CIMA, University of Navarra, IDISNA, Pamplona 31008, Spain; 2 Department of Immunology and Oncology, Centro Nacional de Biotecnología (CNB)-CSIC, Madrid 28049, Spain; 3 Department of Immunology and Biotechnology, Szentágothai Research Center, University of Pécs, Pécs H-7624, Hungary; 4 Division of Molecular Histopathology, Department of Pathology, Cambridge University, Cambridge CB2 1QP, UK; 5 MRC Toxicology Unit and Ernest and Helen Scott Haematological Research Institute, Department of Cancer Studies and Molecular Medicine, University of Leicester, Leicester LE2 7LX, UK; 6 Department of Haematology, Royal Bournemouth Hospital, Bournemouth BH7 7DW, UK; 7 Department of Hematology, Clinica Universidad de Navarra, IDISNA, Pamplona 31008, Spain; 8 Institute of Human Genetics, Christian-Albrechts-University Kiel & University Hospital Schleswig-Holstein, Campus Kiel, Kiel 24105, Germany; 9 Bio-informatic Unit, Department of Genomics and Proteomics, Center for Applied Medical Research CIMA, University of Navarra, IDISNA, Pamplona 31008, Spain; 10 Department of Genetics, School of Medicine, University of Navarra, IDISNA, Pamplona 31008, Spain; 11 Centre for Translation Cell and Tissue Research, KU Leuven, Leuven 3000, Belgium; 12 Department of Pathology, Institut Universitaire du Cancer de Toulouse-Oncopole, Labex TOUCAN and CRCT INSERM U1037, Toulouse F-31053, France; 13 Centro de Biologia Molecular Severo Ochoa, CSIC/Universidad Autonoma, Madrid 28049, Spain; 14 Experimental Therapeutics and Translational Oncology Program, Institute of Molecular and Cellular Biology of Cancer, CSIC/University of Salamanca; and Institute of Biomedical Research of Salamanca (IBSAL), Salamanca 37007, Spain; 15 Molecular Genetics Unit, University Hospital Marques de Valdecilla and IFIMAV, Santander 39011, Spain; 16 Hematology Department, Hospital San Pedro, Logroño, 26006, La Rioja, Spain; 17 Department of Hematology, Complejo Hospitalario de Navarra, Servicio Navarro de Salud, IDISNA, Pamplona 31008, Spain; 18 Department of Pathology, Cancer Research Program, Institut Municipal d'Investigacions Mèdiques (IMIM), Hospital del Mar, Barcelona 08003, Spain; 19 Department of Clinical Hematology, Cancer Research Program, Institut Municipal d'Investigacions Mèdiques (IMIM), Hospital del Mar, Barcelona 08003, Spain; 20 Department of Hematology, ICO-Hospital Universitari Germans Trias i Pujol, Josep Carreras Leukaemia Research Institute, Universitat Autònoma de Barcelona, Badalona 08916, Spain; 21 Department of Hematology, IBSAL-University Hospital and IBMCC-CSIC, University of Salamanca, Salamanca 37007, Spain; 22 Department of Hematology, Hospital Clinico, INCLIVA Biomedical Research Institute, University of Valencia, Valencia 46010, Spain; 23 Department of Pathology, Hospital Clinico, University of Valencia, Valencia 46010, Spain; 24 Department of Medicine/Hematology-Oncology, Weill Cornell Medical College, New York, New York 10065, USA

## Abstract

NKX2 homeobox family proteins have a role in cancer development. Here we show that *NKX2*-*3* is overexpressed in tumour cells from a subset of patients with marginal-zone lymphomas, but not with other B-cell malignancies. While *Nkx2-3*-deficient mice exhibit the absence of marginal-zone B cells, transgenic mice with expression of NKX2-3 in B cells show marginal-zone expansion that leads to the development of tumours, faithfully recapitulating the principal clinical and biological features of human marginal-zone lymphomas. NKX2-3 induces B-cell receptor signalling by phosphorylating Lyn/Syk kinases, which in turn activate multiple integrins (LFA-1, VLA-4), adhesion molecules (ICAM-1, MadCAM-1) and the chemokine receptor CXCR4. These molecules enhance migration, polarization and homing of B cells to splenic and extranodal tissues, eventually driving malignant transformation through triggering NF-κB and PI3K-AKT pathways. This study implicates oncogenic NKX2-3 in lymphomagenesis, and provides a valid experimental mouse model for studying the biology and therapy of human marginal-zone B-cell lymphomas.

## Abstract (web summary)

The homeobox NKX2 family of transcriptional factors has been shown to regulate fundamental developmental processes. Here, the authors show that NKX2-3 is a *bona fide* oncogenic driver in marginal-zone B-cell lymphoma and that it promotes lymphomagenesis by shaping lymphocyte dynamics and promoting BCR signalling.

## Graphical abstract

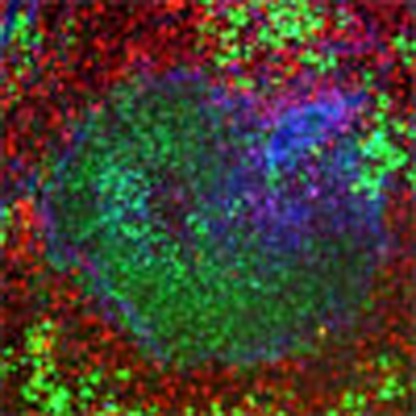

The NKX2 family of orphan homeobox proteins is composed of transcriptional factors that regulate various fundamental cellular processes, including head patterning, cardiac and lung development, and neural cell specification^1^,^2^,^3^,^4^. Several NKX2 members including NKX2-1, NKX2-2 and NKX2-8 function as oncogenic drivers in solid tumours^5^,^6^,^7^,^8^, whereas NKX2-1, NKX2-2 and NKX2-5 frequently show genomic rearrangements leading to deregulated expression in T-cell acute lymphoblastic leukaemia^8^,^9^,^10^. The homeobox NKX2-3 protein controls salivary gland, tooth and small intestine development^3^,^11^,^12^, and has been identified as an important regulator of splenic morphology^12^,^13^,^14^ and vasculature^15^. In fact, NKX2-3-deficient mice (Nkx2-3^−/−^) showed impaired distribution of B, T and follicular dendritic cells in the spleen as a consequence of abnormal homing^12^. However, rather than modulating intrinsic lymphoid development or specification, NKX2-3 directs the splenic micro-environmental conformation to allow normal B-cell maturation, and accordingly, bone marrow reconstitution experiments failed to correct abnormal structures found in the spleens of Nkx2-3-deficient mice^11^,^12^,^13^,^15^,^16^,^17^. In humans, sequence variants in *NKX2*-3 gene predispose to Crohn's disease and ulcerative colitis development, and NKX2-3 expression is upregulated in tissue samples from these patients^18^,^19^,^20^. Beyond these data, little is known about the functional roles of the homeobox protein NKX2-3, and to date it has not been implicated in cancer development^21^.

Chromosomal translocations involving the immunoglobulin (*IG*) gene loci are a hallmark of B-cell lymphomas^22^. Although the molecular study of the common *IG* translocations has led to the discovery of seminal cancer genes such as *MYC*, *BCL2* and *CCND1*, cloning of other less frequent translocations has also identified genes with critical biological functions, such as *BCL9*, *BCL10* and *BCL11A*^23^. We therefore postulated that the study of rare *IG*-related translocations might still pinpoint genes with unappreciated roles in lymphocyte biology and neoplastic transformation. Molecular cloning of the breakpoints of a t(10;14)(q24;q32) chromosomal translocation in a case of B-cell lymphoma identified the *NKX2*-*3* gene in chromosome 10q24.2 juxtaposed to the *IG* heavy-chain (*IGH*) gene in 14q32.33, resulting in increased *NKX2-3* expression. Further quantitative PCR studies revealed increased expression of *NKX2-3* in a subset of patients with extranodal and splenic marginal-zone lymphomas (SMZLs), but not in other B-cell malignancies. Transgenic expression of human NKX2-3 in mouse B cells induced the development of lymphomas recapitulating the principal clinical and biological characteristics of human SMZL. NKX2-3 aberrant expression resulted in constitutive B-cell receptor (BCR) signalling, which in turn activated integrins, adhesion molecules and chemokine receptors that enhanced migration and promoted homing of B cells to splenic and other extranodal tissues, eventually driving malignant transformation. Our study reveals NKX2-3 as a *bona fide* oncogenic driver in marginal-zone B-cell lymphomas, and provides an experimental mouse model to study the functional biology and therapy of this lymphoma entity.

## Results

### *NKX2*-3 expression in marginal-zone B-cell lymphomas

**Figure 1:**
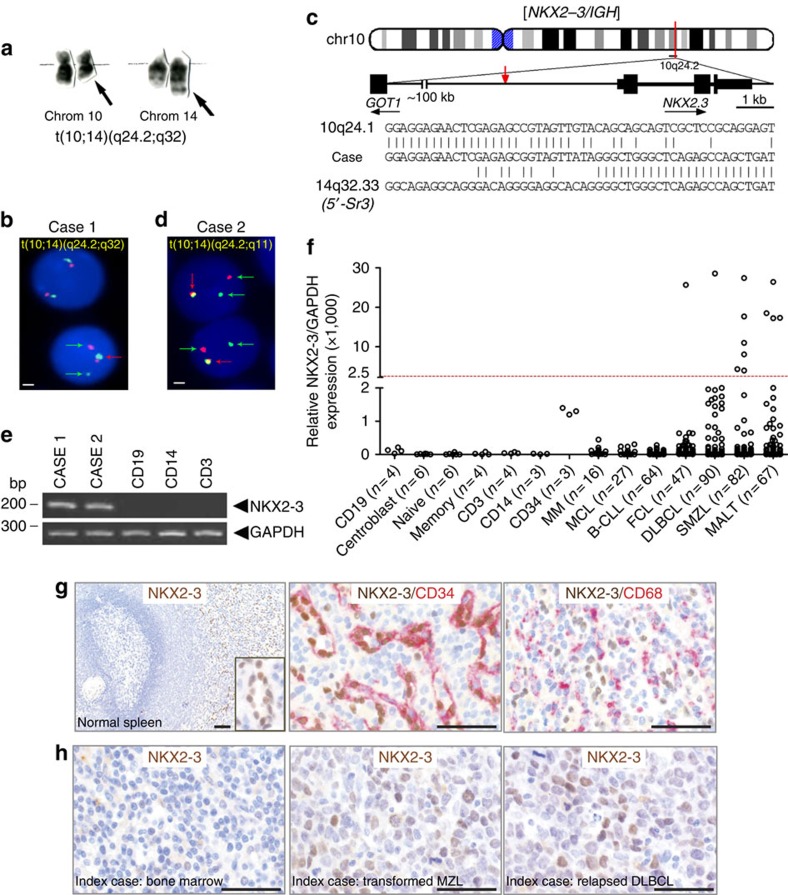
*NKX2-3* expression is deregulated in marginal-zone B-cell lymphomas. (**a**) Partial G-banded karyotype showing a t(10;14)(q24;q32) translocation in a patient with SMZL (case 1). Arrows mark the derivative chromosomes 10 and 14. (**b**,**d**) Interphase FISH analysis of bone marrow cells from two patients with t(10;14) using an *NKX2-3* break-apart assay. Cells carrying the translocation show split of green and red probes (green arrows), in addition to the co-localized signals on the normal allele (red arrows). Scale represents 2 μm in all cases. (**c**) Ideogram depicts location of breakpoints cloned by LDI-PCR from the *IGHS* segment in the t(10;14)(q24;q32) from case 1. Representative breakpoint sequences with identity to *IGHS* and the corresponding *NKX2-3* gene are shown. (**e**) Expression of *NKX2-3* by reverse transcription–PCR cell subpopulations in peripheral blood of healthy donors. (**f**) *NKX2-3* expression measured by quantitative RT–PCR in samples obtained from patients with mature B-cell malignancies and in non-tumoral cells isolated from healthy donors: CD19^+^ B cells, CD3^+^ T cells and CD14^+^ myeloid cells (peripheral blood); pre-germinal centre IgD^+^ naïve B cells, post-germinal center CD27^+^ memory B cells and CD71^+^ germinal centre centroblasts (tonsils); and CD34^+^ haematopoietic stem/progenitor cells (bone marrow). The cutoff value for positive NKX2-3 expression was considered when greater than s.d. (standard deviation) x4 of the mean value of the expression of CD19^+^ cells plus CD34^+^ cells. B-CLL, B-cell chronic lymphocytic leukaemia; DLBCL, diffuse large B-cell lymphoma; FCL, follicular lymphoma; MALT, mucosa-associated lymphoid tissue lymphoma; MCL, mantle cell lymphoma; MM, multiple myeloma; SMZL, splenic marginal-zone lymphoma. The number of patient samples analysed in each experiment is shown. (**g**) IHC analysis of non-tumoral human spleen tissues using moAbs for NKX2-3 (clone 454C/H9), CD34 and CD68. (**h**) IHC analysis of different tissues samples from the case with t(10;14)(q24;q32) (case 1). In the left panel, the bone marrow biopsy obtained at diagnosis shows expression of NKX2-3 in scattered lymphoid B cells. In the middle and right panels, sequential lymph node biopsies obtained during histological transformation to non-GC DLBCL and subsequent relapse display nuclear expression of NKX2-3 in large B-cell lymphoma cells. Scale represents 100 μm in all cases.

Molecular cloning by long-distance inverse PCR (LDI-PCR)^24^ of a chromosomal translocation t(10;14)(q24;q32) in a patient with marginal-zone B-cell lymphoma (case 1) mapped the breakpoints to 3 kb centromeric to the *NKX2*-*3* gene at 10q24.2 and to the 5′-Sγ3 region of *IGH* gene at 14q32.33 (Fig. 1a–c). To ascertain whether the *NKX2-3* gene locus was recurrently targeted by chromosomal translocations, fluorescence *in situ* hybridization (FISH) was used to screen 86 human B-cell lymphoma samples enriched for chromosome 10q22-26 aberrations based on cytogenetic data. Notably, FISH analysis of another B-cell lymphoma carrying a chromosomal translocation t(10;14)(q24;q11) (case 2) showed the juxtaposition of *NKX2*-3 to the gene encoding T-cell receptor alpha (*TCRα*; Fig. 1d). In both lymphomas, increased *NKX2-*3 expression was observed with respect to non-tumoral B lymphocytes (Fig. 1e). Therefore, *NKX2-3* gene expression is deregulated by chromosomal translocations involving antigen receptor loci in B-cell lymphoma.

To delineate the pattern of expression of *NKX2-3* during haematopoietic and lymphoid development as well as in lymphoid neoplasms, quantitative real-time–PCR (qRT–PCR) was performed in different FACS-sorted human cell populations and in a collection of B-cell malignancies (Fig. 1f). Although low levels of *NKX2*-3 expression were detected in human bone marrow-isolated CD34^+^ haematopoietic stem/progenitor cells, expression of *NKX2-3* could not be significantly detected in mature B cells, T lymphocytes or myeloid cells. However, together with the two cases with chromosomal translocations involving the *NKX2-3* locus, increased *NKX2*-3 expression was detected in 12 of 393 samples from patients with untreated B-cell lymphoid malignancies (3%), mainly including 6 of 82 (7%) SMZLs and 4 of 67 (6%) mucosa-associated lymphoid tissue (MALT) lymphomas. On the other hand, *NKX2-3* expression was found in only 2 out of 244 samples (0.8%) from diffuse large B-cell lymphoma (DLBCL), follicular lymphoma, mantle cell lymphoma, chronic lymphocytic leukaemia or multiple myeloma (*P*=0.001; Fisher exact test; Fig. 1f). Immunohistochemistry (IHC) analysis using a novel rat monoclonal antibody (mAb) against the full-length GST-fused human NKX2-3 form (286aa, NM_145285.1) confirmed the previous qRT–PCR data, as human non-tumoral B, T and myeloid cells appeared negative for NKX2-3 expression. However, NKX2-3 expression was selectively detected in the nuclei of splenic CD31^+^vWF^+^CD68^−^CD34^+^αSMA^−^ cells (Fig. 1g), corresponding to endothelial cells in the red pulp sinus^25^. In addition, IHC analysis showed expression of NKX2-3 in the nuclei of lymphoma B cells with the t(10;14)(q24;q32) chromosomal translocation (case 1) as well as in other marginal-zone lymphoma cases (Fig. 1h). Finally, direct sequencing of *NKX2-3*-coding sequence showed no evidence of pathogenic mutations in lymphoma samples (Supplementary Table 1). Overall, these data indicate that NKX2-3 is normally not expressed in mature B lymphocytes, but its aberrant expression is preferentially observed in a subset of patients with marginal-zone lymphomas (including SMZL and MALT lymphomas).

### Characterization of NKX2-3 deficiency in B cells

**Figure 2:**
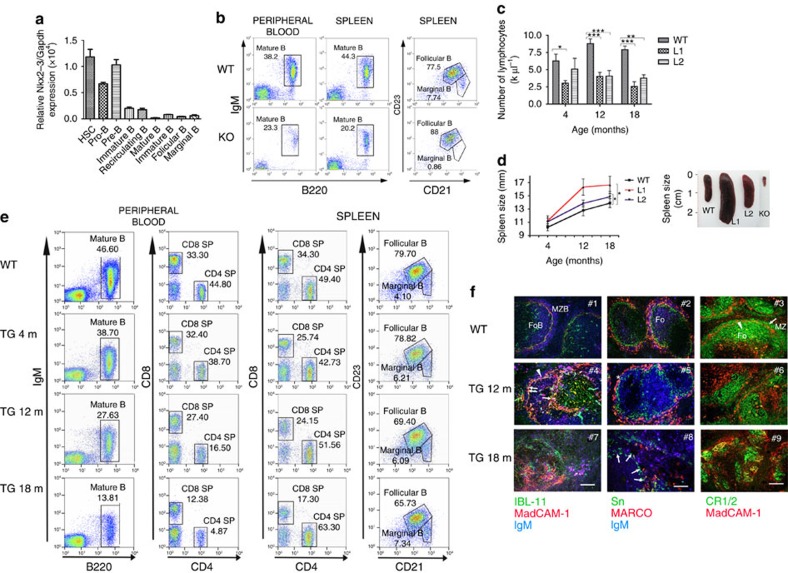
Nkx2-3^−/−^ and Eμ-*NKX2-3* transgenic (TG) mice show abnormal lymphopoiesis. (**a**) Murine *Nkx2-3* expression measured by quantitative RT–PCR in haematopoietic cell subpopulations isolated from healthy C57BL/6 mice. Haematopoietic stem cells (HSC, lin^neg^Sca-1^+^c-Kit^+^), pro-B (c-Kit^+^B220^low^) and pre-B cells (CD25^+^B220^low^) isolated from bone marrow; mature B cells (IgM^+^B220^+^) isolated from peripheral blood; B220^+^CD21^high^CD23^low^ marginal-zone and B220^+^CD21^int^CD23^high^ follicular B cells isolated from the spleen. Three to six mice were used in each experiment. Error bars represent standard deviation (s.d.). (**b**) Representative flow cytometry plots showing different B-cell subpopulations in 8-month-old WT and Nkx2-3^−/−^ mice (KO). (**c**) Sequential lymphocyte cell-count in peripheral blood from WT and TG mice at 4, 12 and 18 months (*n*=10 each). Error bars represent standard deviation (s.d.). Significance level of changes are indicated (two-tailed Student's *t*-test: *P<0.05, **P<0.001, ***P<0.0001). (**d**) Spleen size monitored by ultrasounds measured at 4, 12 and 18 months in WT and TG animals (WT, *n*=8; L1, *n*=10; L2, *n*=12). Spleen images from WT, TG (L1 and L2) and Nkx2-3-deficient (KO) mice. (**e**) Representative flow cytometry plots showing B- and T-cell subpopulations in PB and spleen from 18-month-old WT and 4-, 12- and 18-month-old Eμ-*NKX2-3* mice. (**f**) Immunofluorescence analyses of WT and 12- and 18-month-old TG spleens. Left column: gradual expansion of MAdCAM-1-positive IgM and plasma cells with dissolution of the follicular architecture in ageing Eμ-*NKX2-3* mice: in WT mice (top) marginal reticular cells (arrow, green) adjacent to MadCAM-1 sinus-lining cells (red) separate follicular (FoB) and MZ B cells (MZB, blue, IgM) mice. Middle column: Loss of MZ macrophages in ageing Eμ-*NKX2-3* mice: in WT mice (top) two concentric layers of MZ macrophages consist of an expanded rim of MARCO-positive (red) MZ macrophages encircling sialoadhesin-positive metallophilic macrophages (green) intermingled with IgM-positive MZ B cells (blue; Fo). Right column: dissolution of follicular stromal architecture in ageing Eμ-*NKX2-3* mice: in WT mice (top) CR1/2 receptors (green) mark FDC reticula (arrow) and outline several layers of marginal zone B cells (arrowhead) outside the MadCAM-1 rim of marginal sinus-lining cells (red; Fo, follicle; MZ, marginal zone). m, month.

Consistent with the expression pattern of NKX2-3 in human lymphoid subpopulations, murine *Nkx2-3* was expressed at low levels in isolated bone marrow haematopoietic stem/progenitor cells and in pro-B/pre-B lymphocytes from healthy C57BL/6 mice, but not in more mature B-cell subpopulations (Fig. 2a). To explore the potential role of NKX2-3 during B-cell development, the frequency of different B-cell populations in several lymphoid organs from 4- and 8-month-old Nkx2-3^−/−^ mice was examined. Flow cytometry analysis did not reveal marked differences among B- and T-cell subpopulations in the bone marrow or thymus of Nkx2-3^−/−^ and wild-type (WT) animals (Supplementary Table 2). Therefore, although subtle changes in other minor subcellular fractions cannot be discarded, no evidence of NKX2-3 function at the major immature B-cell stages could be defined. However, a decrease in the total number of B cells was observed in Nkx2-3^−/−^ spleens, including a complete absence of B220^+^CD21^high^CD23^low^ marginal-zone B cells, whereas the B220^+^CD21^int^CD23^high^ follicular B-cell compartment was comparable to WT littermates (Fig. 2b). Furthermore, this dramatic MZ phenotype was accompanied by a moderate reduction of circulating B220^+^IgM^+^ B cells in peripheral blood (PB) of Nkx2-3^−/−^ mice (Fig. 2b). Together, these results support the notion that NKX2-3 may affect splenic marginal-zone organization through regulating homing and distribution of B cells rather than directly affecting B-cell development^11^,^13^.

### *NKX2-3* promotes expansion of splenic marginal-zone B cells

To explore the functional consequences of NKX2-3 expression in B cells *in vivo*, C57BL/6-Tg transgenic mice using the EμSRα enhancer were generated, aiming to drive expression of human *NKX2-3* gene in B lymphocytes, thus mimicking the t(10;14)(q24;q32) in the index case 1. Two independent founder mouse lines (L1 and L2) were characterized (Supplementary Fig. 1a–d). As expected, 2-month-old mice showed restricted expression of the *NKX2-3* transgene in haematopoietic tissues, including CD19^+^ splenic B cells and CD3^+^ T lymphocytes (Supplementary Fig. 1e,f). Although L1 mice showed higher expression of *NKX2*-3 than L2 mice, and exhibited a more pronounced phenotype, both lines showed comparable overall results. In Eμ-*NKX2-3* mice, from about 4 months of age, a progressive reduction in the number of PB lymphocytes accompanied by splenomegaly were observed (Fig. 2c,d and Supplementary Table 3). Sequential flow cytometry studies in mouse haematopoietic cell compartments at 4, 12 and 18 months of age did not find significant changes in the more immature subpopulations in the bone marrow and thymus (Supplementary Table 4). However, a gradual decline in the number of circulating PB mature B220^+^IgM^+^ B lymphocytes and CD4^+^ and CD8^+^ T lymphocytes (including a 3.5-fold decrease in the CD4^+^/CD8^+^ cell ratio) was observed, which became more evident in 18-month-old mice (Supplementary Table 4). Conversely, the total number of B lymphocytes increased ten times in transgenic spleens in comparison with age-matched controls, including a moderate expansion of B220^+^CD21^high^CD23^low^ marginal-zone B cells and a reduction of B220^+^CD21^int^CD23^high^ follicular B cells (Fig. 2e). Immunofluorescence (IF) analysis of the splenic tissue architecture in 12-month-old transgenic mice revealed that IgM^+^MadCAM-1^+^ B cells were detectable, initially at the T:B-cell boundary and the adjacent region between the follicles and marginal zone. These transgenic B cells substantially expanded by 18 months, whereas both the MadCAM-1^+^ marginal sinus and its marginal reticular cell support were destroyed (Fig. 2f). This process was accompanied by a severe disorganization of the marginal-zone macrophage architecture, leading to the near-complete loss of MARCO-positive marginal zone macrophages and a substantial reduction in the number of sialoadhesin-positive metallophilic macrophages. In addition, gradual loss of follicular-stromal architecture (evidenced through fragmentation and collapse of follicular dendritic meshwork) was detected. However, the segregation of white pulp vasculature and red pulp venous sinus network (lost in Nkx2-3^−/−^ mice)^16^ was maintained in Eμ-*NKX2-3* mice (Supplementary Fig. 2). Therefore, expression of NKX2-3 in lymphocytes led to lymphopenia in PB and to a progressive splenomegaly with marginal-zone B-cell expansion, leading to a profound disorganization of the normal splenic architecture. Notably, this picture is opposite to the atrophic spleens with the absence of marginal-zone B cells observed in Nkx2-3^−/−^ mice^11^,^13^.

### Eμ-*NKX2-3* mice develop human-like marginal-zone lymphomas

**Figure 3:**
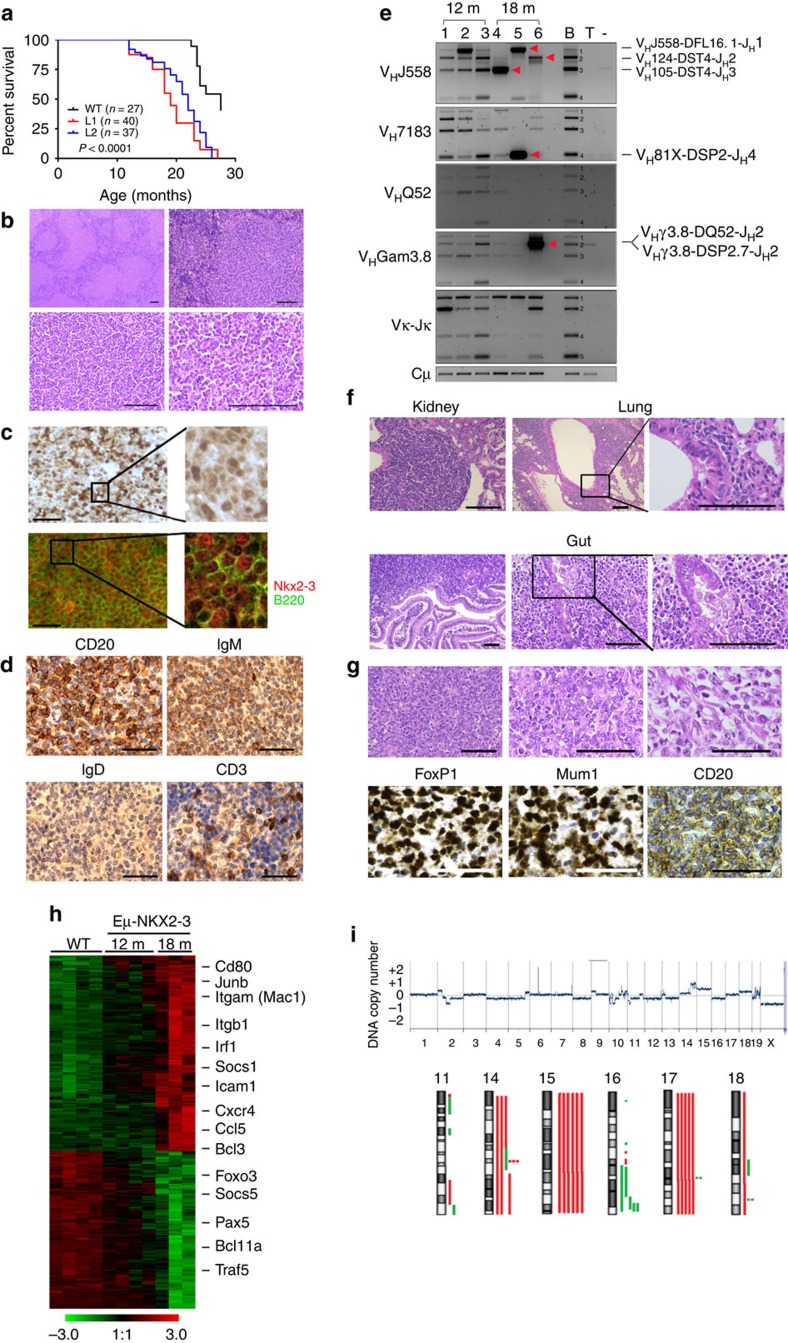
Eμ-*NKX2-3* mice develop human-like marginal-zone lymphomas. (**a**) Kaplan–Meier overall survival curves for the two Eμ-*NKX2-3* transgenic lines and WT mice. (**b**) Haematoxilin-eosin (H&E) staining of formalin-fixed paraffin-embedded spleen tissues of Eμ-*NKX2-3* transgenic mice. Scale represents 100 μm in all cases. (**c**) Immunohistochemical (IHC) and immunofluorescence (IF) studies of transgenic spleens using NKX2-3 and B220 antibodies. (**d**) IHC studies of transgenic spleen biopsies using CD20, IgD, IgM and CD3 antibodies. (**e**) Detection of *Ig* gene rearrangements by PCR on genomic DNA from CD19^+^ cells isolated from transgenic spleens in 12- and 18-month-old mice. *VDJH* clonal rearrangements were identified by direct sequencing (marked with red arrowheads). Approximate sizes for WT B-cell V(D)J rearrangement bands (labelled 1 to 4) are Jh1: 1800, bp, Jh2: 1200, bp, Jh3: 800 bp and Jh4: 300 bp. B and T, healthy murine B (CD19^+^) and T (CD3^+^) lymphocytes, respectively. (**f**) H&E staining of extranodal lymphomas developed in the kidney, the lung and the gut, showing lymphoepithelial lesions. (**g**) H&E staining of spleen tissue biopsies showing transformation areas resembling DLBCL. IHC showed positive staining for using CD20, FoxP1 and Mum1 (Irf4), whereas Gcet1, Bcl10 and Bcl6 expression was not observed. (**h**) Heat-map image of gene expression profiling data showing the 12- and 18-month-old transcriptional signatures in comparison to WT splenic B lymphocytes. (**i**) Whole-genome array-based comparative genomic hybridization (aCGH) analysis of isolated CD19^+^ cells from nine clonal splenic lymphomas developed in 18-month-old transgenic mice. Chromosomal losses and gains are shown in green and red colours, respectively (bottom). A representative example is shown (top). m, month.

Aged Eμ-*NKX2-3* mice showed clinical signs of disease and exhibited shorter survival in comparison to WT animals (median overall survival for L1 and L2 versus WT mice, 17.5 and 20.4 months versus 25.6 months, respectively; *P*<0.0001; Fig. 3a). Upon necropsy, all 38 examined mice (26 from L1, 12 from L2) showed enlarged spleens, whereas 34 mice (89%) also displayed extranodal tumours in the small intestine (*n*=30), the kidneys (*n*=22), the liver (*n*=19), the salivary glands (*n*=7) and the lungs (*n*=6; Supplementary Table 5). Histological analysis of transgenic spleens revealed a prominent white pulp, composed of large B-cell infiltrates and poorly developed T-cell areas, with an almost absence of the red pulp. Compartmentalization of the follicle mantle into a marginal-zone and lymphocytic corona completely disappeared. The mantle-zone was almost entirely populated by small B lymphocytes and clusters of these cells could also be seen in the red pulp, sometimes invading the sinuses (Fig. 3b). IF and IHC studies detected expression of NKX2-3 in the nuclei of tumour B220^+^ B cells, whereas NKX2-3 expression was not detected in WT mouse lymphocytes (Fig. 3c). Immunophenotypic characterization showed that B lymphocytes lacked CD5 expression, and were marked by high surface expression of IgM and low IgD expression (Fig. 3d). Analysis of *Igh*, *Igk* and *Igl* gene rearrangements revealed that 18-month-old splenic B cells harboured clonal rearrangements without *IgVh* somatic hypermutation, but this clonality was absent in younger 12-month-old transgenic mice despite significant splenomegaly being present (Fig. 3e). Collectively, these features are consistent with the diagnosis of human-like SMZL, which in a fraction of cases show unmutated *IGVH* gene sequences^26^,^27^. In addition, tumours within extranodal sites were composed of a heterogeneous population of small and centrocyte-like mature B cells that infiltrated the epithelium and formed lympho-epithelial lesions, mimicking human MALT lymphomas^28^,^29^ (Fig. 3f). Notably, few splenic and extranodal lymphomas (5 of 32 cases, 16%) showed areas containing large B lymphoblasts with high proliferation rates, suggestive of histological transformation to DLBCL, which is a common feature in human marginal-zone lymphomas, including index Case 1 bearing the t(10;14)(q24;q32) translocation^30^,^31^. These mouse DLBCLs showed expression of Irf4 and Foxp1, but were negative for Bcl6, CD10 and Gcet1 staining, consistent with a non-germinal centre origin, which is coincident with the type of DLBCL developed in case 1 (ref. 32; Fig. 3g). Finally, splenic lymphomas could be propagated in immunodeficient Rag2^−/−^IL2γc^−/−^ mice (Supplementary Fig. 3a), demonstrating the malignant potential of transformed NKX2-3-positive cells. Collectively, these data show that Eμ-*NKX2-3* mice generated tumours mirroring the spectrum of human NKX2-3-expressing B-cell lymphomas.

To determine whether mouse and human lymphomas shared molecular attributes, gene expression profiling was performed in NKX2-3-expressing mouse splenic lymphomas and compared with a series of human SMZL samples. Using Linear Models of Microarray Data Analysis, 42 probe-sets were found differentially expressed between CD19^+^ B cells isolated from WT and 12-month-old transgenic spleens, whereas this difference increased to 630 probe-sets between WT and 18-month-old splenic B cells (*B*>0; False discovery rate (FDR)<0.27 and FDR<0.02, respectively; Fig. 3h and Supplementary Data 1). These data indicate that polyclonal B cells from 12-month-old transgenic spleens display subtle gene expression differences compared with normal B lymphocytes, which substantially increase in the clonal splenic lymphomas in older mice. Then, gene expression data from resting CD19^+^ human tonsillar B cells and human SMZL samples were used to define a human SMZL transcriptional signature (*B*>0; FDR<0.03; Supplementary Data 2), which could then be compared to the mouse lymphomas. Unsupervised clustering analysis grouped human SMZL and murine 18-month-old lymphoma samples together (Supplementary Fig. 3b). Accordingly, the human SMZL transcriptional signature appeared overrepresented in the murine 18-month-old lymphoma transcriptional datasets, but not in the 12-month-old lymphoma datasets (hypergeometric test, *P*=0.0098, and *P*=0.51, respectively). Likewise, comparison of the NKX2-3 mouse lymphoma transcriptional signature with two previously published human SMZL gene expression data sets revealed a significant overlap (*P*=0.002 and *P*=0.007; hypergeometric test)^33^,^34^. Finally, microarray-based high-resolution comparative genomic hybridization showed genomic abnormalities in 18-month-old clonal lymphomas (9 of 14, 64%) but not in 12-month-old splenic tumours (*n*=5). These changes targeted chromosomal areas syntenic to regions that were the most frequently rearranged in previous studies of 136 human SMZL biopsies: trisomies of 3q/12q and losses of 7q/8p (Fig. 3i and Supplementary Table 6)^35^,^36^. Overall, these data show that Eμ-*NKX2-3* splenic lymphomas and human SMZLs share significant histopathological, immunohistochemical, molecular and genomic features.

### NKX2-3 induces constitutive BCR signalling in young mice

**Figure 4:**
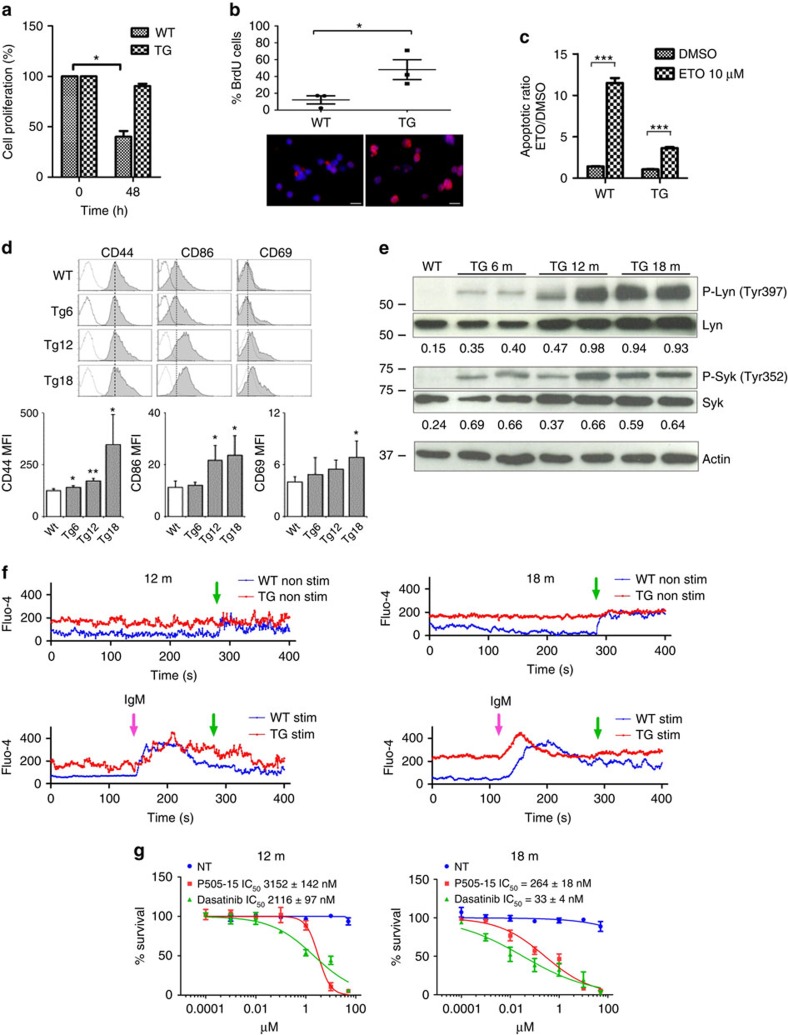
NKX2-3 induces B-cell receptor signalling in B cells from young mice. (**a**) Cell survival of *in vitro* cell cultures containing isolated CD19^+^ cells from WT and transgenic (TG) mice. Data from three experiments are shown. (**b**) BrdU staining of WT and TG splenic CD19^+^ cells isolated after 20 h of BrdU administration. A representative merged DAPI (blue) and BrdU (red) staining image is shown (bottom). Results of BrdU quantification in WT and TG mice are shown (top). Scale represents 10 μm in all cases. (**c**) Apoptosis quantification after 48 h of incubation with etoposide (ETO) or dimethylsulphoxide (DMSO) in B cells from WT and NKX2-3 TG mice. Data from three experiments are shown. Error bars represent standard deviation (s.d.). The statistical significance levels of the changes are indicated (two-tailed Student's *t*-test: *** *P*<0.0001). (**d**) Top, Representative flow cytometry profiles (grey filled histogram) of the indicated molecules at the surface of WT B cells and NKX2-3 TG B cells of the specified ages (Tg6, 6-month-old mice, Tg12, 12-month-old mice and Tg18, 18-month-old mice). Dashed black line, maximum expression levels in WT B cells. Dotted histogram, isotype control. Bottom, quantification of the mean fluorescence intensity (MFI) for CD44, CD86 and CD69 surface expression in WT and Tg B cells of the indicated ages; data are the mean±s.d. (*n*=3 mice in each case). (**e**) Western blot analysis showed increased phosphorylation levels of Syk and Lyn kinases in CD19^+^ splenic B cells isolated from TG mice at 6, 12 and 18 months. Quantification of phosphorylated Lyn and Syk proteins was determined by densitometry, indicating the ratio respect to the non-phosphorylated form. (**f**) Kinetics of calcium mobilization in splenic B cells from WT and TG NKX2-3 mice at 12 and 18 months. Top and bottom graphs show the kinetics of non-stimulated and stimulated cells with goat anti-mouse IgM, respectively. Pink arrows indicate the time points of the stimulation (stim.), whereas green arrows indicate the time points of addition of Ca^2+^-containing buffer. (**g**) Survival of splenic Eμ-*NKX2-3* CD19^+^ B-cell lymphoma cell cultures incubated with the Syk and Lyn protein inhibitors (P505-15 and Dasatinib, respectively) at increasing concentrations. Non-treated cells (NT) were incubated with DMSO. IC_50_, half maximal inhibitory concentration. Error bars indicates s.d.

Virtually all human SMZL and MALT lymphomas show constitutive BCR activation, which is triggered through variable mechanisms including microbial antigenic stimulation or mutations in BCR signalling components^27^,^28^,^37^. These lymphomas largely depend on BCR signalling for their survival^38^. To test whether NKX2-3-induced mouse lymphomas showed constitutive BCR signalling, B220^+^ B cells isolated from transgenic and WT spleens were analysed. *In vitro*, transgenic B cells exhibited prolonged cell survival, increased proliferation (as shown by higher 5-bromodeoxyuridine (BrdU) incorporation rates), and reduced apoptosis (shown by Annexin/PI staining) in comparison to WT B cells (Fig. 4a–c). In addition, flow cytometry analysis revealed an activated cell phenotype (CD44^+^, CD69^+^, CD86^+^), which was increasingly evident in an age-dependent manner (Fig. 4d). Transgenic B cells showed surface expression of IgM and IgD, without age-related significant changes (Supplementary Fig. 4a). Investigation of the functional status of the BCR by western blot analysis revealed phosphorylation of Lyn (Tyr397) and Syk (Tyr352) tyrosine kinases in NKX2-3-expressing B cells, indicating constitutive BCR signalling (Fig. 4e). This abnormal activation was already detected in 6-month-old mouse B lymphocytes, becoming progressively more evident in 12-month-old tumours and in 18-month-old splenic lymphomas. Accordingly, increased and more persistent basal levels of calcium mobilization were observed in transgenic B cells, either without stimulation or under anti-IgM stimulation conditions (Fig. 4f). Moreover, tumour cells exhibited reduced surface expression of CD22 and of PD-1, and diminished SHP-2 phosphatase activity in comparison to WT cells (Supplementary Fig. 4b,c). Importantly, although survival of NKX2-3 transgenic B cells from younger animals was moderately impaired after incubation with Lyn and Syk inhibitors, this impairment was significantly greater in 18-month-old clonal B-cell lymphoma cells (IC_50_ within low nano-molar ranges; Fig. 4g). These data indicate that NKX2-3-expressing B cells are prone to exhibit an activated phenotype and prematurely trigger BCR signalling in young mice even before developing clinical evidence of disease. Furthermore, constitutive BCR activation appears increasingly evident with time, and transgenic B cells become progressively dependent on BCR signalling for survival.

**Figure 5:**
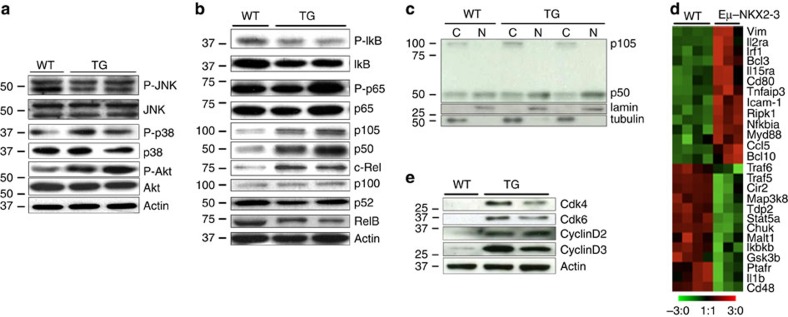
NKX2-3 induces activation of BCR and downstream signalling pathways in mouse B-cell lymphomas. (**a**) Western blot analysis showed increase in AKT phosphorylation levels in splenic CD19^+^ cells isolated from 18-month-old transgenic (TG) mice in comparison to WT cells, whereas similar levels of phosphorylation of Jnk (p36/p54) and p38 proteins were detected. (**b**) Western blot analysis showed similar RelB and p100/p52 expression in transgenic and WT cells, excluding activation of non-canonical NF-κB pathway. However, expression of p50 and of c-Rel proteins was higher in 18-month-old B-cell lymphoma cells in comparison to WT cells, indicating triggering of the canonical NF-κB pathway. Two representative transgenic and WT CD19^+^ splenic B cells are depicted. (**c**) Western blot analysis of p105 and p50 proteins in cytoplasmic (C) and nuclear (N) extracts from WT and 18-month-old transgenic CD19^+^ splenic cells. (**d**) Heat-map image showing gene components of the NF-κB pathway and NF-κB target genes (http://www.bu.edu/nf-kb/gene-resources/target-genes) differentially expressed between 18-month-old splenic B-cell lymphoma cells and WT splenic CD19^+^ B lymphocytes. (**e**) Western blot analysis of cell cycle proteins in splenic CD19^+^ cells isolated from WT and transgenic mice.

It is known that constitutive BCR signalling in human SMZL cells commonly activates downstream NF-κB pathways, and less frequently alternative MAPK, NFAT and AKT/mTOR pathways^27^,^28^,^39^,^40^. To determine whether similar events occurred in the NKX2-3-induced splenic lymphomas, investigation of the status of the BCR downstream signalling cascades was performed. Western blot analysis revealed phosphorylation of AKT, leading to PI3K-mTOR activation, but not of other MAP kinases including JNK and p38 (Fig. 5a). In addition, activation of the canonical NF-κB cascade (as shown by higher expression levels of nuclear p50—pointing to an increased processing of p105- and of c-Rel proteins) but not of the alternative NF-κB pathway was detected (Fig. 5b,c). Canonical NF-κB signalling increased the expression of gene components of the NF-κB pathway and NF-κB target genes (*P*<0.000001) in the 18-month-old transcriptional lymphoma data set, and also induced overexpression of NF-κB target proteins (Fig. 5d,e). Super-shift assays carried out using antibodies against NF-κB subunits showed a shift of p50 band in transgenic B cells, thus confirming the previous findings (Supplementary Fig. 5a). Of note, although Lyn and Syk phosphorylation were detected in 6-month-old transgenic B cells, activation of PI3K-AKT or NF-κB signalling were detected in 18-month-old clonal lymphoma cells but not in younger mice, demonstrating them to be late events during lymphoma development (Supplementary Fig. 5b,c). Overall, these data suggest that chronic activation of BCR signalling is an early event in the transformation of NKX2-3-expressing mature B cells, whereas constitutive activation of downstream PI3K-AKT and NF-κB pathways appears to be late events potentially contributing to lymphomagenesis.

### NKX2-3 regulates B cells dynamics in lymphoid tissues

**Figure 6:**
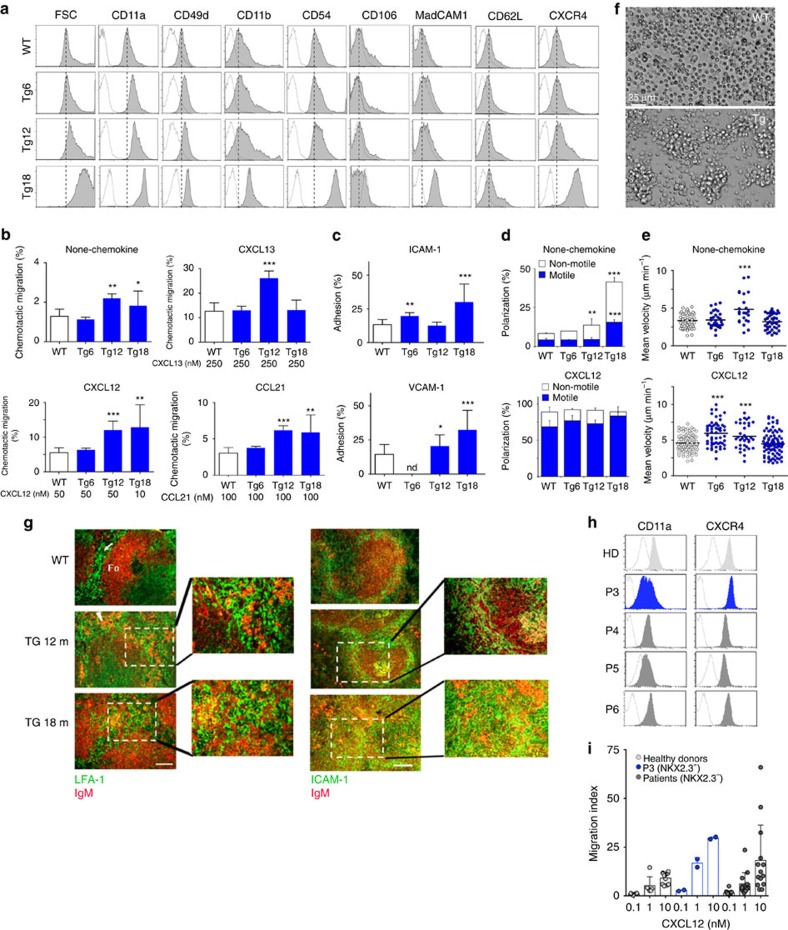
Phenotypic analysis and characterization of cell dynamics in NKX2-3-expressing B cells. (**a**) Representative flow cytometry profiles (grey filled histogram) of forward side scatter (FSC) and the indicated molecules at the surface of WT B cells and NKX2-3 transgenic (Tg) B cells of the specified ages (Tg6, 6-month-old mice, Tg12, 12-month-old mice and Tg18, 18-month-old mice). Dashed black line, maximum expression levels in WT B cells. Dotted histogram, isotype control. (**b**) Migration frequencies of WT and transgenic B cells of the indicated ages in Boyden chambers in the absence or presence of CXCL12, CXCL13 or CCL21. Data are the mean±s.d. (*n*=4). Error bars represent standard deviation (s.d.) in all cases. (**c**) Frequency of adhesion to ICAM-1 (top) or VCAM-1 (bottom) containing artificial membranes of WT and transgenic B cells. Data are the mean±s.d. (*n*=2). (**d**) Cell polarization frequencies, indicating the fractions of non-motile and motile cells, of WT and transgenic B cells settled on ICAM-1-membranes in the absence (top) or presence of CXCL12 chemokine (bottom); data are the mean±s.d. (*n*=2). (**e**) Mean velocity values of motile WT and transgenic B cells; data are derived from two experiments and each dot is a single cell. (**f**) Representative wide-field images of WT B cells (top) and transgenic B cells (bottom) after 24 h of culture. (**g**) IF analysis of WT and transgenic spleens from 12- and 18-month-old mice: (left) Clustering of B cells in LFA-1^+^ compartments in ageing Eμ-*NKX2-3* mice: in WT mice (top) LFA-1^+^ (green) cells are restricted to the red pulp (arrow), whereas the follicles stained with anti-IgM (red; Fo) are devoid of such cells. Scale bar, 200 μm. (**h**) Representative flow cytometry profiles (filled histogram) of the indicated molecules at the surface of human B cells from healthy donors (HD) and patients (P3, patient positive for NKX2-3 expression; P4, P5, P6, patients negative for NKX2-3 expression). Dotted histogram, isotype control in each case. (**i**) Migration index of human B cells from healthy donors (*n*=4), P3 (patient positive for NKX2-3) and patients negative for NKX2-3 expression (*n*=7) in response to gradients of CXCL12.

In normal and malignant B cells, BCR signalling regulates lymphocyte dynamics by modulating their traffic and tissue location^41^. Moreover, previous studies of Nkx2-3^−/−^ mice here and elsewhere already pointed towards a critical role of NKX2-3 in the organization of the marginal-zone area and the lymphocyte homing in the spleen, involving the deregulation of chemokines and cell adhesion proteins such as MadCAM-1 (refs 11, 12, 13, 14, 15). To test whether this was the case in the Eμ-*NKX2-3* mouse model, flow cytometry analysis for relevant homing makers was applied to isolated transgenic and WT CD19^+^ B cells. Remarkably, increased expression of multiple cell surface proteins implicated in homing and cell motility was detected in the tumour cells, including integrins such as LFA-1 (CD11a), VLA-4 (CD49d) and MAC-1 (CD11b), and adhesion molecules such as MadCAM-1, ICAM-1 (CD54) and L-selectin (CD62L). In addition, augmented expression of the chemokine receptor CXCR4, but not of CXCR5 or CCR7, was detected in transgenic B cells (Fig. 6a and Supplementary Fig. 6a). Like BCR signalling, these phenotypic changes occurred gradually, being detected from 12 months of age and thereafter. However, these were not observed in younger mice. Functional studies of cell adhesive and motile capacities revealed that transgenic B cells displayed a significant and progressive increase in migration, either without stimuli or in the presence of CXCL12 (CXCR4 ligand), CCL21 (CCR7 ligand) and CXCL13 (CXCR5 ligand) gradients (Fig. 6b). Next, assessment of integrin activation and cell adhesion showed that, in the absence of stimuli, lymphoma cells increased their adhesion to both ICAM-1- and VCAM-1-containing substrates (Fig. 6c, Supplementary Fig. 6b), indicating basal activation of LFA-1 and VLA-4 integrins, respectively. Then, using a two-dimensional model based on artificial planar lipid bilayers containing GPI-linked ICAM-1 (ref. 42), B-cell dynamics was analysed in real time. Although no differences were observed in young mice (Supplementary Movie 1), a significant enhancement in cell polarization (membrane protrusion activity) and motility was observed in 12-month-old and older transgenic B cells, which moved with higher mean velocity values and described longer tracks than WT B lymphocytes (Fig. 6d,e and Supplementary Movies 2 and 3). NKX2-3-expressing B cells also moved faster in response to CXCL12 or CXCL13 chemokines (Fig. 6e, Supplementary Fig. 6c and Supplementary Movies 4–6). Finally, transgenic cells adhered more strongly between them in comparison to WT B lymphocytes, forming large clusters *in vitro* (Fig. 6f and Supplementary Movies 3–6). To better visualize cell cluster formation *in vivo*, IF analysis of transgenic spleen sections was performed. A progressive development of small-to-medium sized clusters composed of LFA-1- and ICAM-1-expressing cells intermingled with IgM-producing cells was documented in 12-month-old tumours, reflecting that the cluster formation was facilitated by adhesion molecules and integrins that favoured adhesion of B cell among themselves and with surrounding environmental cells, promoting the development of large non-clonal tumour masses. Eventually, in 18-month-old spleens, clonal tumours increased in size and completely disorganized the splenic architecture (Fig. 6g).

To ascertain whether B cells from patients with SMZL with expression of NKX2-3 showed similar homing features to those observed in Eμ-*NKX2-3* murine splenic lymphomas, eight PB samples from patients with untreated SMZL and from six healthy donors were analysed. Using qRT–PCR, one of the eight SMZL samples showed expression of *NKX2-3*. Interestingly, these P3 lymphoma cells exhibited higher expression levels of LFA-1 and CXCR4 in comparison to *NKX2*-3-negative SMZL samples (Fig. 6h). Furthermore, and consistently with our previous results in mice (Fig. 6b), B cells from P3 lymphoma also showed an increased migration index to CXCL12 with respect to the other samples (Fig. 6i). These results indicate that human SMZL with expression of NKX2-3 partially recapitulates the features of NKX2-3-induced lymphomas in mice.

### NKX2-3-expressing B cells are sensitive to BCR blockade

**Figure 7:**
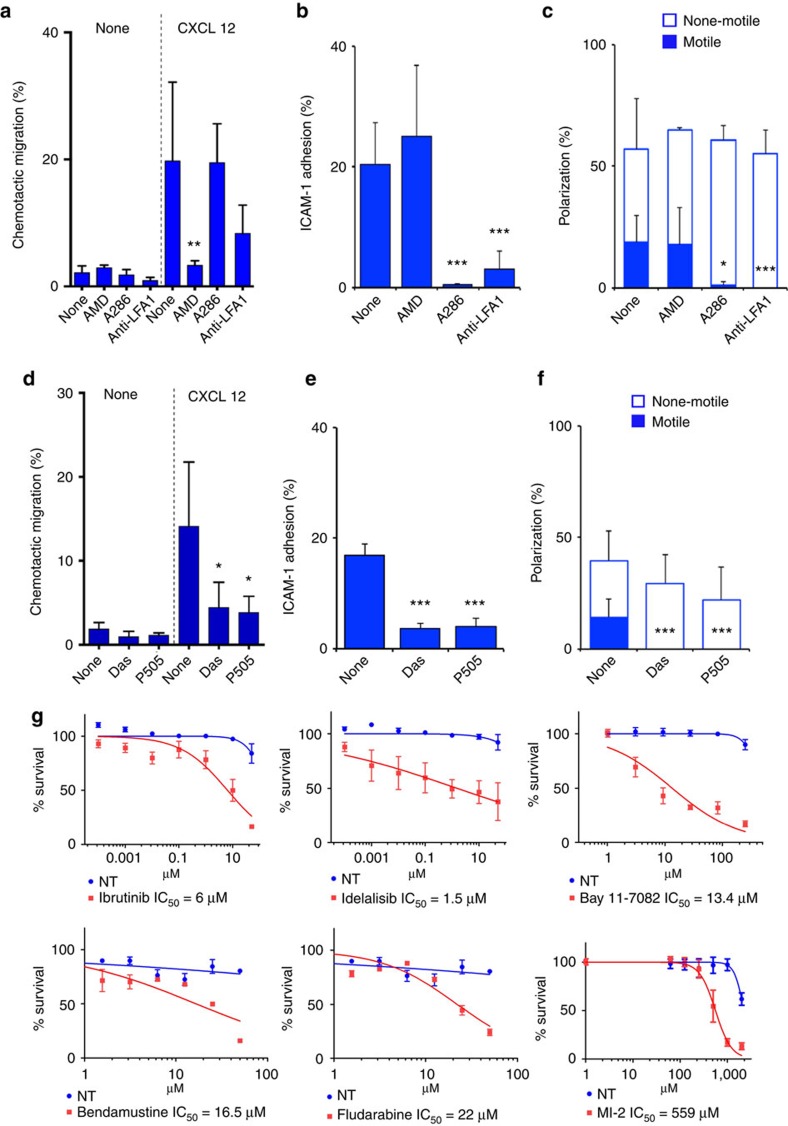
Functional characterization of cell dynamic changes in *E*μ*-*NKX2-3 transgenic mice. (**a**) Migration frequencies of transgenic B cells non-treated (none) or treated with the indicated chemical inhibitors (AMD, CXCR4 antagonist, 10 μM; A286, LFA-1–ICAM-1 interaction inhibitor, 10 μM; anti-LFA1, blocking antibody anti-mouse CD11a, 1 μg ml^−1^) in Boyden chambers in the absence (none-chemokine) or presence of CXCL12 (10 nM). Significance levels of changes are indicated (two-tailed Student's *t*-test: *P<0.05, **P<0.001, ***P<0.0001) in all **a**–**f** cases. (**b**) Frequency of adhesion to ICAM-1-containing artificial membranes of transgenic B cells none-treated or treated with the indicated chemical inhibitors. (**c**) Cell polarization frequencies, indicating the fractions of non-motile and motile cells, of transgenic B cells none-treated or treated with the indicated chemical inhibitors and settled on ICAM-1-membranes in the absence of CXCL12 chemokine. Data in **a**,**b**,**c** are the mean±s.d. (*n*=4). (**d**) As in **a** using the chemical inhibitors Dasatinib (Das, 1 μM; Lyn inhibitor) and P505-15 (P505, 1 μM; Syk inhibitor). (**e**) As in **b** using the indicated inhibitors. (**f**) As in **c** using the indicated inhibitors. Data in **d**,**e**,**f** are the mean±s.d. (*n*=3). (**g**) Survival of splenic Eμ-*NKX2-3* CD19^+^ B-cell lymphoma cell cultures incubated during 24 h with the BTK inhibitor (Ibrutinib), the PI3K inhibitor (Idelalisib), the MALT1 proteolytic inhibitor (MI2), the NF-kB inhibitor (Bay11-7082), bendamustine and fludarabine at increasing concentrations. Non-treated cells (NT) were incubated with dimethylsulphoxide (DMSO). IC_50_, half maximal inhibitory concentration. Error bars indicates s.d.

Next, to determine the molecules responsible for the observed changes in lymphocyte dynamics, transgenic B-cell lymphoma cells were incubated with different chemical inhibitors. Although no major differences were observed in cell migration in the absence of stimuli, in the presence of CXCL12 gradients, migration of B cells was almost completely abrogated with a selective CXCR4 antagonist (AMD3100), but not with compounds blocking LFA-1 function (A286982; anti-LFA1 antibody; Fig. 7a). Conversely, adhesion of transgenic B cells to ICAM-1-containing substrates was markedly reduced by blocking LFA-1 or ICAM-1–LFA-1 interactions, but not upon CXCR4 inhibition (Fig. 7b). Similarly, cell motility was selectively impaired with ICAM-1/LFA-1 inhibitors but not with the CXCR4 antagonist, while cell polarization was not affected (Fig. 7c). Then, whether BCR signalling contributed to the changes in lymphocyte dynamics was investigated by incubating transgenic B cells with non-lethal doses of Lyn and Syk chemical inhibitors. A partial reduction in Lyn/Syk phosphorylation levels was associated with the reversion of the observed phenotype, including the abrogation of cell migration in the presence of CXCL12 gradients, adhesion to ICAM-1 membranes and abnormalities in cell motility (Fig. 7d–f). Overall, these data show that NKX2-3-induced BCR signalling increased the expression of multiple surface molecules in transgenic B cells, including CXCR4 that increased cell migration, whereas ICAM-1 and LFA-1 enhanced cell adhesion and motility. These cells abnormally homed to secondary lymphoid tissues, leading to progressive B-cell accumulation and eventually driving malignant transformation.

Finally, we used the NKX2-3-induced mouse lymphomas to evaluate the potential therapeutic efficacy of drugs targeting the BCR and downstream signalling molecules. Treatment with the PI3K inhibitor Idelalisib (IC_50_, 1.5 μM), the BTK inhibitor Ibrutinib (IC_50_, 6 μM), the MALT1 proteolytic inhibitor MI2 (IC_50_, 0,56 μM), the NF-kB inhibitor Bay11-7082 (IC_50_, 13.4 μM) and two drugs currently used in the treatment of SMZL (bendamustine and fludarabine) reduced B-cell lymphoma cell survival, but to a much lesser extent than the chemical inhibitors of Lyn (Dasatinib, IC_50_, 33 nM) and Syk (p505-15, IC_50_, 264 nM) kinases (Fig. 7g). These data suggest that targeting the BCR with Lyn and Syk inhibitors may be of therapeutic value in patients with marginal-zone lymphoma.

## Discussion

This study reveals that *NKX2-3* is targeted by *IG*-related translocations and is abnormally expressed in a subset of patients with extranodal and SMZLs, but very rarely in other B-cell malignancies. Although several members of the NKX2 homeobox family have been involved in the development of solid tumours and T-cell leukaemias^8^,^21^, ectopic expression of NKX2-3 in mouse B lymphocytes induced the development of clonal B-cell neoplasms closely mirroring the spectrum of human NKX2-3-expressing lymphomas. These findings define the homeobox NKX2-3 family protein as a *bona fide* oncogenic driver in marginal-zone B-cell lymphomagenesis in human and mice.

A better understanding of the cellular and molecular mechanisms underlying the development of marginal-zone lymphomas will facilitate the diagnosis and treatment of this heterogeneous disease^27^,^37^,^43^. Currently, there are few diagnostic and prognostic markers for patients with marginal-zone lymphomas, and there is not a clear molecular target for these tumours, particularly for SMZL. In addition, despite the fact that there are animal models mimicking the principal features of human MALT lymphoma^44^, such models are lacking for SMZL. We propose that the Eμ-*NKX2-3* mice fully recapitulate the main cellular, histopathological, molecular and genetic features of human SMZL, thus representing a valid tool for studying the functional biology and therapy of this lymphoma entity. The fact that lymphomas from these Eμ-*NKX2-3* mice exhibit activation of the BCR pathway is particularly interesting, because virtually all patients with marginal-zone lymphomas show constitutive BCR signalling, which is triggered by different inflammatory and molecular mechanisms^45^. However, in a number of cases, the cause of BCR activation remains elusive. We show that in a small fraction of tumours, NKX2-3 plays a role in activating BCR signalling, which may be through a direct effect on Lyn/Syk tyrosine kinases. However, it is also possible that alternative indirect mechanisms, for instance antigenic stimulation, are required during the transformation process. Indeed, we have used the MD4 BCR transgenic mouse model (in which all B cells express a BCR specific for hen egg lysozyme) to evaluate the putative role of BCR activation by autoantigens in the tumour development process. Analysis of three NKX2-3 transgenic mice MD4 negative (polyclonal BCR repertoire) and three transgenic mice NKX2-3 MD4 positive (monoclonal BCR repertoire) at 6 months of age showed lower levels of activated Syk in the latter group in comparison to the MD4-negative mice. These data suggest that antigen stimulation of the BCR may have a role in the lymphomagenesis observed in our mouse model (Supplementary Fig. 4d).

Eμ-*NKX2-3* mice exhibited a sequential model of lymphoma development. Constitutive BCR signalling was observed from 6 month of age, which was followed by significant molecular and cellular dynamic changes in B cells that facilitated their homing to spleen tissues, whereby they became adherent and were retained by stromal cells to accumulate and progressively develop non-clonal tumours. At ∼18 months, B cells acquired genomic rearrangements that activated NF-κB and PI3K-AKT signalling pathways, which promoted cell proliferation and survival, progressively inducing the formation of clonal B-cell lymphomas. Although constitutive activation of BCR and downstream signalling pathways has been implicated in human marginal-zone lymphoma development^34^,^39^,^40^, abnormal migration, homing and adhesion as mechanisms involved in the pathogenesis of these tumours represent novel findings that warrant further investigation. Intriguingly, many patients with SMZL are preceded by an asymptomatic low-count B-cell lymphocytosis that progressively increases in number and in some cases associates with splenomegaly, eventually fulfilling the diagnostic criteria of SMZL^46^. Accordingly, this observation suggests that human SMZL and NKX2-3-induced mouse lymphomas may follow a similar sequential model of tumour development. Although our model recapitulates many aspects of human marginal-zone lymphoma biology, we think it has limitations that include the long tumour latency (which on the other hand reflects the slow development of SMZL observed in patients), and the fact that tumours are triggered by the activation of NKX2-3 in B cells, which is only observed in a fraction of patients with MALT lymphoma and SMZL.

In summary, our study reveals that oncogenic NKX2-3 promotes B-cell lymphomagenesis by activating BCR signalling and disturbing lymphocyte dynamics, and provides an experimental mouse model that recapitulates the major features of human SMZL. Finally, our data suggest that targeting the BCR with inhibitors of Lyn and Syk kinases may be of therapeutic value in patients with marginal-zone lymphomas.

## Methods

### Human primary samples

A patient diagnosed of marginal-zone lymphoma showed an abnormal karyotype in 4 out of 16 examined bone marrow cells: 46,XY,del(7)(q32q36),t(10;14)(q24;q32). DNA isolated from these cells was used to clone the t(10;14)(q24;q32) by LDI-PCR^24^. In addition, four-hundred eighty samples obtained from untreated patients with mature B-cell malignancies, collected from different institutions participating in the study, were analysed. The study was performed in the accordance with the regulations of the Institutional Review Board of the University of Navarra, and was conducted according to the Declaration of Helsinki principles. Informed consent was obtained from all patients.

### Long-distance inverse PCR

LDI-PCR was performed to screen for rearrangements involving the *IGHJ* segments in the lymphoma cells carrying the t(10;14)(q24;q32). Briefly, high-molecular-weight DNA was digested with restriction enzymes chosen from the results of DNA blot to yield small DNA IGHJ fragments. Phenol/chloroform extraction was performed to remove residual enzymatic activity, and 0.4 μg of digested DNA was then ligated at 15 °C overnight in a total volume of 500 μl with 5 U of T4 DNA ligase (Promega). The ligated DNA was then purified using the Wizard DNA clean-up system (Promega) and eluted in a final volume of 40 μl. Primers were designed within the *JH* and *IGH* enhancer regions based on published sequences (Supplementary Table 7), so that the amplification products would include short stretches from these regions in addition to the rearranged or translocated regions 5′ of *JH*. In addition, restriction sites to permit forced cloning into plasmid vectors were introduced^24^.

### Quantitative real-time PCR

Total RNA was isolated from harvested cells and patient samples using TRIzol (Invitrogen). RNA was converted into cDNA using random hexanucleotides and the M-MLV reverse transcriptase (Invitrogen). In human samples, *NKX2-3* expression was measured using a TaqMan probe (Hs00414553_g1, Applied Biosystems) normalized to *GAPDH* (Hs99999905_m1, Applied Biosystems). In mouse samples, *Nkx2-3* gene expression quantification was performed using the SYBR Green PCR Master Mix (Applied Biosystems) normalized to mouse *Gapdh*. Quantitative real-time–PCR was performed in the 7300 Real-Time PCR System with universal PCR Master Mix (Applied Biosystems). For each sample, a ΔCt value was calculated as the threshold cycle (Ct) value of the *NKX2-3/Nkx2-3* gene (target) minus the Ct value of *GAPDH/Gapdh* (control). A cutoff value for considering a sample positive for *NKX2-3* expression was calculated as four-times the standard deviation (s.d.) of the 2^−ΔCt^ value of NKX2-3 expression in the human CD19^+^ and CD34^+^ cell samples. The primers used are listed in Supplementary Table 7.

### Fluorescence *in situ* hybridization

FISH was applied to screen for chromosomal breakpoints involving the *NKX2-3* gene locus (chr10: chr10:101,292,690-101,296,280, hg19) using a break-apart assay consisting of two differently labelled BAC clones CTD-3188C19 (chr10:101075221-101281059, hg19) and RP11-157O7 (chr10:101308986-101475976 hg19)^47^, following previously reported methods^48^. Screening for *NKX2-3* gene abnormalities was performed on fixed cells left over from routine cytogenetic studies of 86 mature B-cell leukaemias and lymphomas, including 37 cases with chromosomal changes affecting bands 10q22-q26, 39 cases with other cytogenetic abnormalities, and 10 cases with a normal karyotype. The *NKX2-3-TCRA/D* fusion was investigated using a triple colour assay, which included the *NKX2-3* probes described above labelled in spectrum aqua and the commercial LSI TRA/D Dual Color Break apart Rearrangement Probe (Abbott/Vysis).

### Generation of NKX2-3 mAb

A human *NKX2-3* cDNA clone encoding full-length NKX2-3 protein was used to bacterially express a GST fusion protein for immunization in two Wistar rats, using previously reported methods^49^. The hybridoma fusion generated seven mAbs, two of which were selected because they selectively reacted with NKX2-3 and not with other NKX2 family proteins by ELISA. Both showed a similar nuclear staining pattern in splenic endothelial cells by IHC, but did not work for western blot analysis. The NKX2-3 5GAL-454C/H9 clone was selected for further studies.

### IHC and western blot analyses

Paraffin-embedded mouse and human lymphoma tissues included in the study were centrally reviewed by at least two expert haemopathologists (listed as authors in this study), and diagnosed according to the World Health Organization criteria^29^. For comparative studies, age-matched WT mice were used. For western blot analysis, equal amounts of total protein (10–50 μg) were separated on SDS–polyacrylamide gel electrophoresis, and electrotransferred onto nitrocellulose membranes. Membranes were incubated with primary antibodies, followed by secondary antibodies conjugated to horseradish peroxidase, which were detected by chemiluminescence (Applied Biosystems and Pierce, respectively)^50^. IHC and western blot antibodies are listed in Supplementary Table 8. In the figures, molecular-weight size markers in kDa are shown. When indicated, the quantifications of bands from western blot was performed by densitometry, and subsequent analysis using Quantity One software (Bio-Rad).

### Generation and characterization of NKX2-3-deficient mice and Eμ-NKX2-3 transgenic mice

Knock-out mice for *Nkx2.3* gene have been previously reported^11^. Human NKX2-3 cDNA was cloned into the *Hin*dIII *Sac*I of the pEμSR vector (kindly provided by Jerry Adams), to obtain the pEμSR-NKX2-3 vector. A 4.455-bp *Pci*I *Aat*II fragment containing the *NKX2-3* gene was isolated from the pEμSR-NKX2-3 vector, purified and injected into fertilized oocytes of C57BL/6 mice background. Mice were crossed onto C57BL/6 genetic background and housed in the specific pathogen-free animal facilities of our centre. All the experiments were conducted with protocols approved by the Ethical Committee of Animal Experimentation of the University of Navarra. Mice were characterized for transgene integration by PCR and Southern blot (Supplementary Fig. 1c,d). The PCR was designed to detect the transgene random integration event. The transgene-containing mice showed a PCR product of 586 bp. (Supplementary Fig. 1c). Mice positive by PCR for the presence of the transgene were verified by Southern blot analysis using a 473-bp. JMC-1 probe designed by PCR amplification from the pEμSR-NKX2-3 vector. The hybridization signal of JMC-1 probe indicated the presence of the *Sac*I fragment in the sample, containing the transgene. Supplementary Fig.1d shows the results of the Southern blot analysis performed in eight animals, allowing the selection of the founder mice used in this study. Primers for the genotyping PCR and the probe construction are listed in Supplementary Table 7. *NKX2-3* transgenic females were crossed with MD4 BCR transgenic males (kindly provided by Dr Yolanda R. Carrasco), both in C57BL/6 background and of similar ages (2–3 months old). The F1 generation was genotyped, and born NKX2-3 transgenic MD4-positive mice in comparison to born NKX2-3 transgenic MD4-negative mice were monitored for B-cell activation and/or transformation.

### Mouse blood cell count

PB samples were collected from transgenic and WT mice. The following parameters were measured using 40 μl of PB with a HEMAVET HV950FS multispecies hematology instrument (Drew Scientific, Inc.): white blood cells, lymphocytes, neutrophils, monocytes, eosinophils, basophils, red blood cells and haemoglobin.

### Measurement of spleen size using ultrasounds

Ultrasound examination of the spleen size was performed in WT and transgenic mice at 4, 12 and 18 months. For each age group, at least eight mice were analysed. The sagital and longitudinal spleen lengths were measured by micro-ultrasound imaging using the Vevo770 high-resolution *in vivo* micro imaging system (VisualSonics), and data were analysed using the Vevo770 v.3.0 software.

### Flow cytometry analyses

BM, PB, spleen, thymus and lymph node nucleated cells were extracted from Nkx2-3^−/−^, Eμ*-NKX2-3* and matched healthy C57BL/6 mice. Preparation of the cells for flow cytometry and staining studies was performed according to the standard procedures^44^,^50^. Cells were acquired in a cytometer FACScalibur (BD Biosciences), and further analysed using the FlowJo 7.6.3 software. Monoclonal antibodies were obtained from BD Biosciences, except for anti-MadCAM-1 antibody (Santacruz Biotechnology), for anti-mouse CCR7 antibody, anti-mouse CD22 and anti-mouse CD279 (PD-1; BioLegend), and for anti-mouse IgM (Jackson ImmunoResearch), and are listed in Supplementary Table 9.

### Cell isolation

Cell sorting of different subpopulations in C57BL/6 mice was performed using a FACSAria (BD Biosciences), as reported^44^. The haematopoietic stem/progenitor cell isolation was performed by incubating BM cells with anti-Sca1 and anti-lineage marker antibodies (CD4, CD8, B220, Gr1 and Mac1) to obtain Sca1^+^Lin^−^ cells. The remaining BM cell subpopulations were marked as follows: pro-B cells, B220^low^c-Kit^+^; pre-B cells, B220^low^CD25^+^; recirculating B cells, B220^high^IgM^+^ and immature B cells, B220^low^IgM^+^. Splenic cell subpopulations were marked as follows: mature B cells, B220^+^IgD^high^IgM^low^, immature B cells, B220^+^IgD^low^IgM^high^, follicular B cells, B220^+^CD23^+^CD21^low^ and marginal-zone B cells, B220^+^CD23^−^CD21^high^. Isolation of splenic CD19^+^ and CD3^+^ cells from WT and transgenic mice were carried out using the AutoMACs separation system with CD19 or CD3 microbeads (Miltenyi-Biotec), respectively. Isolated cell subpopulations were reanalysed for purity by flow cytometry (purity >98%). For chemotaxis and cell dynamics assays, primary B cells from spleens of WT and NKX2-3 transgenic mice were isolated by negative selection (>95% purity) using pan-T (Thy1.2) dynabeads (DYNAL), as described^42^. Age-matched WT and NKX2-3 transgenic mice were used on each experiment. Human B cells were obtained from PB of healthy donors (buffy coats samples kindly supplied by the Centro de Transfusiones Comunidad de Madrid) and patients by negative selection (>95% purity) using Dynabeads Untouched Human B cells kit (DYNAL) after a Ficoll-gradient centrifugation step.

### Cell survival and proliferation

Spontaneous cell survival was determined by incubating isolated WT and transgenic splenic CD19^+^ B cells at a density of 2.5 × 10^6^ cells per ml during 48 h. Cell viability was assessed by trypan-blue dye exclusion. Apoptosis was measured by Annexin V/propidium iodide staining after treatment with etoposide at 10 μM during 48 h. For BrdU staining, cells were fixed with 4% paraformaldehyde, washed with PBS and incubated in 1 N and 2 N HCl, 10 min each, at 37 °C. After PBS washing, cells were treated 12 min with 0.1 M Borate Buffer, and incubated overnight with BrdU mAb following incubation with secondary anti-rat antibody.

### *V(D)J* Recombination analysis of mouse B-cell tumours

*Ig* gene rearrangements were amplified by PCR using primers specific for several V gene families in conjunction with reverse J primers^51^. PCR bands were cloned into a plasmid vector, and eight to ten clones from each purified band were sequenced and analysed for the presence of somatic hypermutation of *Ig* gene sequences.

### Gene expression microarray data analysis

Samples were processed according to reported methods^44^,^50^. The cDNAs were hybridized to the Affymetrix HG-U133 Plus2 arrays in the case of human samples. Mouse samples were similarly processed and hybridized to the Affymetrix GeneChip Mouse Gene 1.0 ST arrays. Raw gene expression microarray data files were submitted to GEO and are available under the accession number GSE49356. Both background correction and normalization were done using RMA (Robust Multichip Average) algorithm. Then, a filtering process was performed to eliminate low-expression probe sets. R/Bioconductor was used for preprocessing and statistical analysis. Linear Models for Microarray Data were used to find out the probe sets that showed significant differential expression between experimental conditions. Genes were selected as significant using a *B* statistic cutoff *B*>0.

### Functional and pathway analysis of gene expression signatures

These analyses were performed according to standard procedures^44^,^50^. Briefly, functional enrichment analysis of Gene Ontology categories was carried out using standard hypergeometric test. The biological knowledge extraction was complemented through the use of Ingenuity Pathway Analysis (Ingenuity Systems, www.ingenuity.com), which database includes manually curated and fully traceable data derived from literature sources. To study the biological meaning of the results, enrichment tests with respect to MsigDB gene sets was carried out. To this end, the non-parametric Kolmogorov–Smirnoff rank test was used as implemented in the Gene Set Enrichment Analysis software. Owing to the stochastic nature of this procedure, the test was performed five times and the *P*-values for each gene-set were computed based on 5,000 permutation iterations. The FDR was calculated for multiple hypothesis testing correction. A hypergeometric test was used to calculate the significance of the overlap between the NKX2-3 signatures and previously published human SMZL transcriptional data set^33^,^34^.

### High-resolution comparative genomic hybridization (aCGH) to microarray

Genomic DNA was extracted from CD19^+^ cells isolated from transgenic (*n*=19) and WT spleens (*n*=2, used as normal hybridization controls). Whole-genome analysis was conducted using a 180-K oligonucleotide mouse aCGH microchip (AMADID 27411, Agilent Technologies), following standard protocols^52^. Microarray data were extracted and visualized using Feature Extraction v10.7 and Agilent Genomic Workbench v5.0 softwares (Agilent Technologies). Regions with DNA copy number abnormalities were detected using ADM-2 (seta s 6) statistic provided by DNA Analytics, with a minimum number of 5 consecutive probes. Genomic build mm7 was used for the experiment. For comparison of the murine lymphoma genomic changes with those present in human disease, two previously published studies including the aCGH analysis of 136 samples from patients with SMZL were used^35^,^36^.

### Chemotaxis assays

Purified B cells (3 × 10^5^) resuspended in 100 μl RPMI 10% FCS were added to the upper insert of Boyden chambers (Costar). Then, the upper insert was placed in the lower chamber that contains 600 μl RPMI 10% FCS alone or in the presence of chemokine (recombinant human CXCL12 and recombinant murine CXCL12, CXCL13, or CCL21; Preprotech) at the specified concentration. Cells were incubated at 37 °C for 2 h 30 min, collected from the lower chamber, and counted for 1 min at high flow rate in a FACScalibur cytometer (BD Bioscience). We use Boyden chambers of 3 μm (mouse B cells) or 5 μm (human B cells) pore size; each assay was done per duplicate. Migration frequency was calculated as the percentage of cells from the input that migrate to the lower chamber ((no. of cells at lower chamber/no. of cells at the input) × 100); the input cell number was obtained placing 100 μl of the initial cell suspension in 500 μl PBS and counting them as explained. In the assays with human B cells, Migration Index was calculated as the ratio between the no. of cells at the lower chamber in each chemokine condition and the no. of cells at the lower chamber in the absence of chemokine (basal migration) for each sample (healthy donor or patient).

### Calcium mobilization assay

Splenic cells (10^7^) from WT and transgenic Eμ-NKX2-3 mice were stained with anti-CD19. Cells were twice washed with RPMI-1640 medium and stained with 2 μM of Fluo-4 AM (Invitrogen) for 30 min. Cells were collected and resuspended in 1 ml of RINGEN buffer (155 mM NaCl; 4.5 mM KCl; 3 mM MgCl_2_; 10 mM D-Glucose; 5 mM HEPES). When indicated, cells were stimulated with 10 μg ml^−1^ anti-IgM, and later, the buffer was supplemented with 2 mM of CaCl_2_. Intracellular Ca^2+^ mobilization was measured in real-time by FACScalibur (BD Biosciences) with a laser tuned at 488 nm. Raw data files were analysed using FlowJo 7.6.4 software.

### Adhesion assays and time-lapse microscopy on planar lipid bilayers

Artificial planar lipid bilayers containing GPI-linked mouse ICAM-1 or VCAM-1 (density 150 molecules per μm^2^) were prepared and used in the study^42^,^53^. Briefly, membranes were assembled on FCS2 chambers (Bioptechs), blocked with PBS/2% FCS (1 h, room temperature (RT)) and when indicated, coated with 100 nM chemokine immediately before use (30 min, RT). Purified NKX2-3 transgenic and WT B cells were fluorescently labelled with CFSE or SNARF-1 probes (0.1 μM, 10 min, 37 °C; Molecular Probes), mixed at 1:1 ratio, injected into the warmed chamber (4 × 10^6^, 37 °C), allowed to settle on the membranes (5–10 min), and imaging was initiated. Confocal fluorescence, differential interference contrast and interference reflection microscopy (IRM) images were acquired every 8 s for 15 min to evaluate cell dynamics; two or three consecutive videos were acquired in distinct membrane positions per chamber. For cell adhesion measurements, snapshots in different membrane positions were acquired. Assays were performed in PBS/0.5% FCS/0.5 g l^−1^ D-glucose/2 mM MgCl_2_/0.5 mM CaCl_2_. Images were acquired on an Axiovert LSM 510-META inverted microscope with a × 40 oil immersion objective (Zeiss). Imaging analysis of cell adhesion and dynamic parameters were done using Imaris 7.0 software (Bitplane) and ImageJ software (NIH). Adhesion frequency was calculated as (no. of cells in contact with the ICAM-1- or VCAM-1-containing membrane (IRM^+^)/total no. cells in the field) × 100; we calculated polarization and motility frequencies similarly. Assays were performed using NKX2-3 transgenic and WT B cells isolated from mice of same age, and comparing data between them. Similar data were obtained with WT B cells independently of the mouse age.

### IF studies

Frozen and acetone-fixed sections were blocked with 5% BSA in PBS for 20 min. Triple label fluorescence for marginal reticular cells, marginal sinus and B cells was performed using FITC-conjugated rat mAb IBL-11 in a cocktail with Cy3-labelled anti-MAdCAM-1 mAb (clone MECA-367) and Alexa Fluor 647-labelled anti-rat IgM (clone B7.6). Three-colour staining for marginal zone macrophages was performed with incubating the sections first with anti-sialoadhesin mAb MOMA-1 (AbD Serotec) in conjunction with FITC-labelled sheep anti-rat IgG (BD Biosciences), where residual binding sites of secondary antibody were blocked with 5% normal rat serum. Subsequently, a cocktail of Cy-labelled anti-rat MARCO (clone IBL-12) and Alexa Fluor-647-labelled anti-IgM was added. Follicular dendritic cells and marginal sinus were identified using a cocktail of FITC-conjugated anti-CD21/35 mAb (clone 7G6 from BD Biosciences) and Cy3-labelled anti-MAdCAM-1 mAb. Staining for VCAM-1 and ICAM-1 expressed by B cells was performed by incubating sections first with unlabelled rat mAbs (anti-VCAM-1 clone 429 from BD Biosciences or anti-ICAM-1 clone YN1/1, kindly provided by Dr Andras K. Szakal) followed by FITC-labelled sheep anti-rat IgG (BD Biosciences) and saturation with 5% normal rat serum. Rat anti-mouse LFA-1 antibody (clone M17/4 from American Type Culture Collection) was used as hybridoma supernatant. B cells were identified using Alexa Fluor 647-labelled anti-rat IgM, for 45 min incubation each at room temperature in a humid chamber. Triple staining for red pulp vessels, marginal sinus and white pulp vasculature was accomplished using a cocktail of FITC-conjugated rat mAb IBL-7/1 against marginal sinus and white pulp vessels, Cy3-conjugated anti-MAdCAM-1 mAb and Alexa Fluor647-labelled IBL-9/2 mAb against red pulp sinuses. After mounting, the sections were viewed under an Olympus BX61 fluorescent microscope. The acquisition of digital pictures with a CCD camera was performed using the analySIS software. IF antibodies are listed in Supplementary Table 8.

### Chemical inhibitors

For therapeutic experiments, mouse transgenic B-cell lymphoma cells were incubated at 2.5 × 10^6^ cells per ml for 24 h with the following compounds at increasing concentrations: PI3K inhibitor (Idelalisib; Chemie Tek); BTK inhibitor (Ibrutinib; Chemie Tek); MALT1 proteolytic inhibitor (MI2); NF-kB inhibitor (Bay11-7082; Enzo LifeSciences); Lyn inhibitor (Dasatinib; Selleckchem); Syk inhibitor (P505-15; Selleckchem). For chemotactic and cell dynamics assays, mouse B cells were incubated at 3 × 10^6^ cells per ml for 1 h at 37 °C with the following compounds: CXCR4 antagonist (AMD3100; 10 μm); blocking rat anti-mouse LFA-1 (CD11a) antibody (clon M17/4; 1 μg ml^−1^; BioLegend); LFA-1–ICAM-1 interaction inhibitor (A286982; 10 μm); Lyn inhibitor (Dasatinib; 1 μm) and Syk inhibitor (P505-15; 1 μm). Then, they were used in the assays keeping the presence of the chemical inhibitor.

### Electrophoretic mobility gel shift array

Cells were lysed and nuclear fractions were resuspended in 20 mM HEPES, pH 7.9, 420 mM NaCl, 1 mM EDTA, 1 mM ethyleneglycol tetraacetic acid and 20% glycerol. Nuclear extracts (10 mg of total protein) were incubated with a 32p-labelled double-stranded DNA probe corresponding to the consensus NF-κB site (5′ -GGGAATTTCC-3′). Samples were run on a 5% non-denaturing polyacrylamide gel in 200 mM Tris-borate, 2 mM EDTA. Gels were dried and visualized by autoradiography. Supershifts were performed using rabbit polyclonal antibodies specific for p50, p65, c-Rel, p52, RelB or irrelevant anti-GATA1 antibodies (all from Santa Cruz Biotechnology).

### Statistical analysis

Overall survival in transgenic mice was defined as the time interval between the date of birth and the date of death. Survival curves were estimated using the product limit method of Kaplan–Meier and were compared using the log-rank test. Two-tailed *t*-tests (normally distributed data) or Mann–Whitney *U*-test (non-normally distributed data) were used for evaluating the significance of differences at gene expression of WT versus transgenic samples, as appropriate. Statistical analyses were carried out using GraphPad Prism 5.0 software (GraphPad). In the case of chemotaxis, cell adhesion and other cell dynamics parameters, statistical analysis and graphs were done using GraphPad Prism 4.0 software. Two-tailed unpaired Student's *t*-test was applied to compare each NKX2-3 transgenic B cells population with its control (WT B cells); *P<0.05, **P<0.001, ***P<0.0001.

## Additional information

**Accession codes:** Affymetrix GeneChip Mouse Gene 1.0 ST gene expression microarray data files have been deposited in the public GEO database and are available under the accession number GSE49356.

**How to cite this article:** Robles, E. F. *et al.* Homeobox NKX2-3 promotes marginal-zone lymphomagenesis by activating B-cell receptor signalling and shaping lymphocyte dynamics. *Nat. Commun.* 7:11889 doi: 10.1038/ncomms11889 (2016).

## Supplementary Material

Supplementary Information Supplementary Figures 1-7 and Supplementary Tables 1-9

Supplementary Movie 1 Dynamics of WT and NKX2-3 transgenic B cells from 6 month-old mice. Caption: DIC and IRM images of SNARF-1-labelled WT B cells (red) and CFSE-labelled NKX2-3 transgenic B cells (green), mixed in 1:1 ratio, in contact with ICAM-1 membranes, over time (15 min; 3 frames/sec) are shown. Tracks are highlighted with dragon tail (red, WT B cells; green, NKX2-3 transgenic B cells).

Supplementary Movie 2 Dynamics of WT and NKX2-3 transgenic B cells from 12 month-old mice. DIC and IRM images of SNARF-1-labelled WT B cells (red) and CFSE-labelled NKX2-3 transgenic B cells (green), mixed in 1:1 ratio, in contact with ICAM-1 membranes, over time (15 min; 3 frames/sec) are shown. Tracks are highlighted with dragon tail (red, WT B cells; green, NKX2-3 transgenic B cells).

Supplementary Movie 3 Dynamics of WT and NKX2-3 transgenic B cells from 18 month-old mice. DIC and IRM images of SNARF-1-labelled WT B cells (red) and CFSE-labelled NKX2-3 transgenic B cells (green), mixed in 1:1 ratio, in contact with ICAM-1 membranes, over time (15 min; 3 frames/sec) are shown. Tracks are highlighted with dragon tail (red, WT B cells; green, NKX2-3 transgenic B cells).

Supplementary Movie 4 Dynamics of WT and NKX2-3 transgenic B cells from 6 month-old mice in presence of CXCL12. DIC and IRM images of SNARF-1- labelled WT B cells (red) and CFSE-labelled NKX2-3 transgenic B cells (green), mixed in 1:1 ratio, in contact with ICAM-1 membranes coated with CXCL12, over time (15 min; 3 frames/sec) are shown. Tracks are highlighted with dragon tail (red, WT B cells; green, NKX2-3 transgenic B cells).

Supplementary Movie 5 Dynamics of WT and NKX2-3 transgenic B cells from 12 month-old mice in presence of CXCL12. DIC and IRM images of SNARF-1- labelled WT B cells (red) and CFSE-labelled NKX2-3 transgenic B cells (green), mixed in 1:1 ratio, in contact with ICAM-1 membranes coated with CXCL12, over time (15 min; 3 frames/sec) are shown. Tracks are highlighted with dragon tail (red, WT B cells; green, NKX2-3 transgenic B cells).

Supplementary Movie 6 Dynamics of WT and NKX2-3 transgenic B cells from 18 month-old mice in presence of CXCL12. DIC and IRM images of SNARF-1- labelled WT B cells (red) and CFSE-labelled NKX2-3 transgenic B cells (green), mixed in 1:1 ratio, in contact with ICAM-1 membranes coated with CXCL12, over time (15 min; 3 frames/sec) are shown. Tracks are highlighted with dragon tail (red, WT B cells; green, NKX2-3 transgenic B cells).

Supplementary Data 1 List of the differentially expressed genes in 18 months Em-NKX2-3 vs. wild-type using LIMMA (B>0, FDR<0.02; 630 genes) defining the Em-NKX2-3 transcriptional signature.

Supplementary Data 2 List of the differentially expressed genes in nine biopsies from SMZL patients vs. human CD19+ cells using LIMMA (B>0, FDR<0.03), defining the SMZL transcriptional signature.
