# Supplementary material for: Homeobox NKX2-3 promotes marginal-zone lymphomagenesis by activating B-cell receptor signalling and shaping lymphocyte dynamics
Source: Nat Commun. 2016 Jun 14;7:11889. doi: 10.1038/ncomms11889 (PMC4911677; doi:10.1038/ncomms11889)
Supplement: Supplementary Information — Supplementary Figures 1-7 and Supplementary Tables 1-9 [file ncomms11889-s1.pdf]

<u>SUPPLEMENTAL MATERIAL</u>

Homeobox NKX2-3 promotes marginal-zone lymphomagenesis by activating B-cell receptor signaling and shaping lymphocyte dynamics

<u>SUPPLEMENTARY FIGURES AND FIGURE LEGENDS</u>

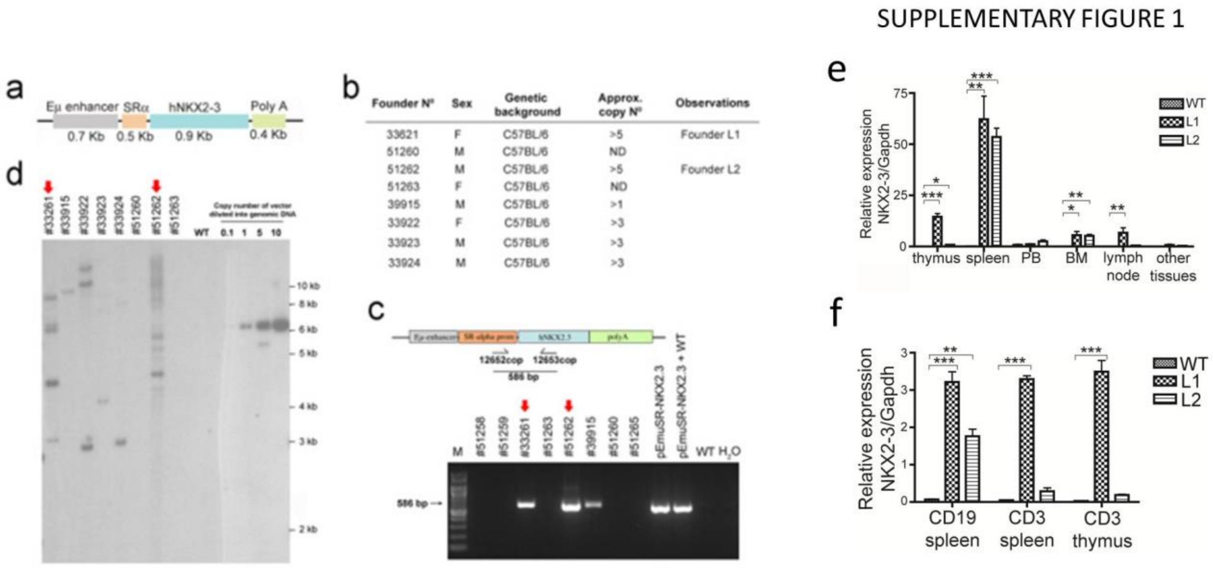

Supplementary Figure 1: Generation and characterization of transgenic Eμ-*NKX2-3* mice. (a) Schematic representation of the EμSRα-hNKX2-3 transgenic vector used to generate *Eμ-NKX2-3* mice. (b) Two founder mice generated (F0) were selected for generating L1 and L2 transgenic lines. (c) Schematic representation of the PCR strategy for transgenic mouse genotyping. PCR results of eight mice are shown. (d) Southern-blot analysis using the JMC1-A probe in transgenic mice. Red arrows in (c) and (d) mark those mice that were selected as founders in the study. (e-f) Expression of the *hNKX2-3* transgene measured by quantitative RT-PCR in WT and transgenic mice in (e) BM, PB and lymphoid tissues; and (f) isolated splenic $CD19^+$ and $CD3^+$ cells, and thymic $CD3^+$ cells.

Supplementary Figure 2

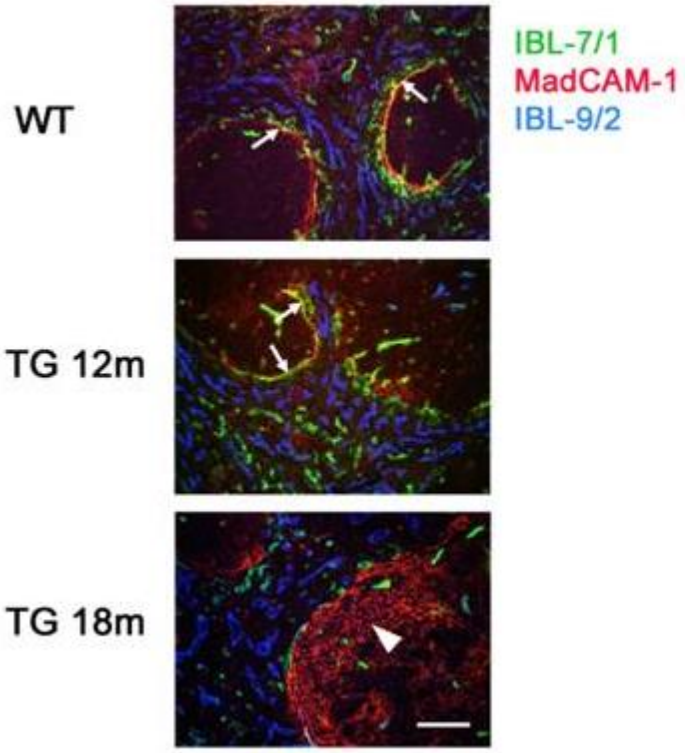

Supplementary Figure 2: Immunofluorescence analysis of *Eμ-NKX2-3* transgenic spleens. Gradual loss of marginal sinus integrity in mice after 12 months is revealed through the loss of IBL-7/1 marker (green) from the marginal sinus (red [arrowheads in upper and middle panels]) and expansion of MAdCAM-1-positive follicular stromal reticulum (arrow in lower panel), with preserved red pulp vessels displaying IBL-9/2 marker[1] (blue). Scale bar = 200 μm.

Supplementary Figure 3

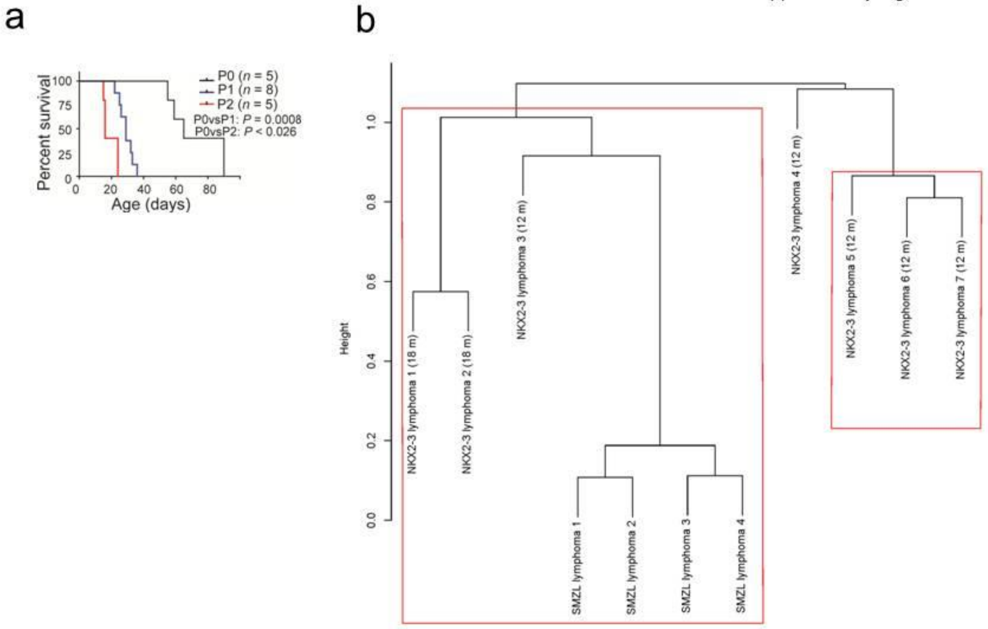

Supplementary Figure 3: Characterization of transgenic Eμ-*NKX2-3* mice. (a) Kaplan-Meier overall survival curves for immunodeficient Rag2$^{-/-}$IL2γc$^{-/-}$ mice intravenously injected with 2.5x10^{6} splenic NKX2-3-expressing lymphoma cells from 18-month-old mice (P0). Survival curves for secondary (P1) and tertiary (P2) recipients are also shown. (b) Dendrogram from a hierarchical clustering analysis of the samples. Clusters with p<0.05 are shown in red.

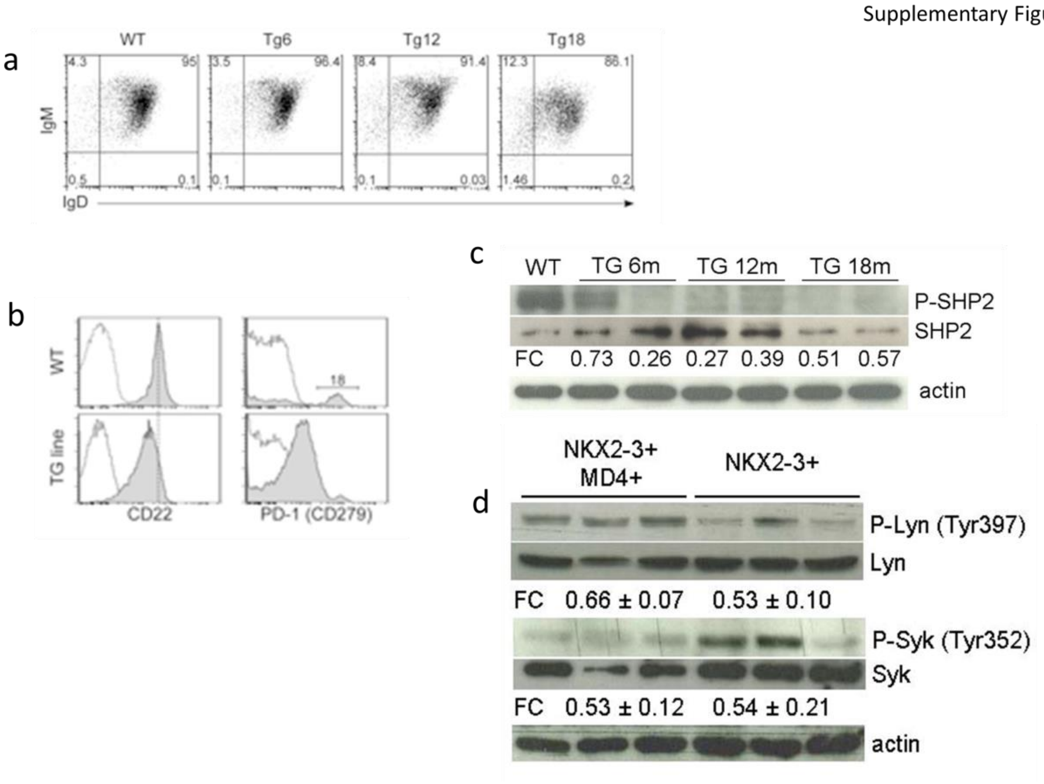

Supplementary Figure 4: Expression and activity of BCR and of BCR signaling-related negative regulators in NKX2-3 transgenic B cells. (a) Representative flow cytometry dotted plots of IgM and IgD expression levels at the surface of WT B cells and NKX2-3 transgenic (Tg) B cells (gated as $CD19^{+}$) of distinct ages (Tg6, Tg12, and Tg18 are 6, 12, and 18-month-old mice, respectively). (b) Representative flow cytometry profiles (grey filled histogram) of CD22 and PD-1 (CD279) at the surface of WT B cells and of an established NKX2-3 transgenic tumoral B cell line (TG line). Dotted histogram, isotype control. Dashed black line, maximum CD22 expression level in WT B cells. In PD-1 profile of WT B cells, the percentage of $PD\text{-}1^{+}$ B cells detected is indicated. (c) Western blot analysis of phosphorylated SHP2 and total SHP2 proteins in wild-type and transgenic mice (d) Western blot analysis of phosphorylated Syk and Lyn kinases in NKX2-3 transgenic mice MD4 negative (polyclonal BCR repertoire) and three transgenic mice NKX2-3 MD4 positive (monoclonal BCR repertoire) at 6 months of age. These studies showed lower levels of activated Syk in the latter group in comparison to the MD4 negative mice, therefore suggesting that antigen stimulation of the BCR may have a role in the lymphomagenesis observed in our mouse model.

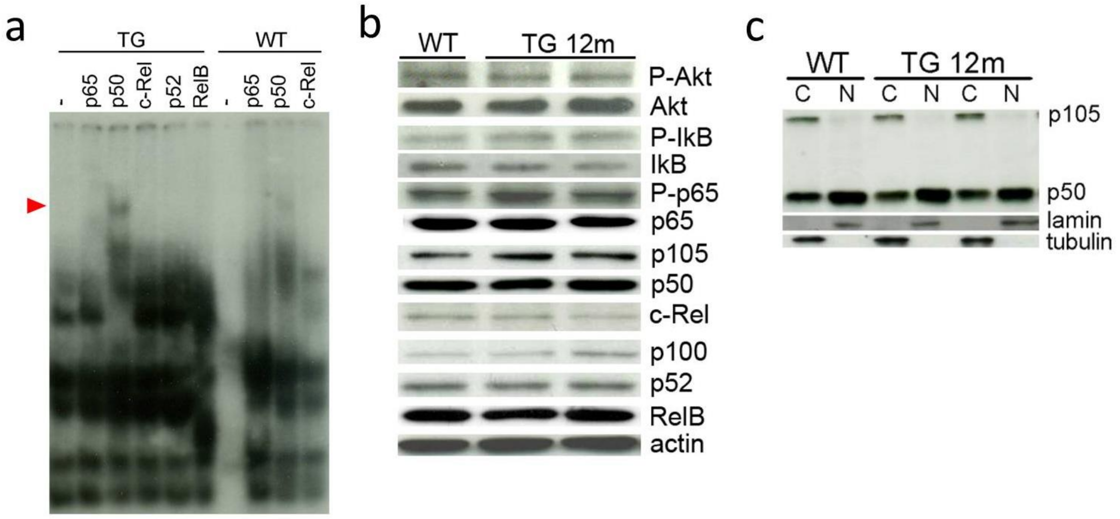

Supplementary Figure 5: Analysis of NF-κB pathway activation in 12-month-old transgenic B cells. (a) Protein-DNA interaction analyzed by electrophoretic mobility shift assay (EMSA) in nuclear extracts of $CD19^+$ cells isolated from transgenic and WT spleens, using a radiolabelled NF-κB consensus probe. Supershifts were performed using canonical and no-canonical antibodies. To induce NF-κB signaling, WT cells were stimulated in presence of anti-CD40 for 24 h. (b) Western-blot analysis of splenic CD19+ cells isolated from WT and 12-months-old transgenic (TG) mice. (c) Western blot analysis of p105 and p50 proteins in cytoplasmic and nuclear extracts from WT and 12-months-old transgenic (TG) $CD19^+$ splenic cells.

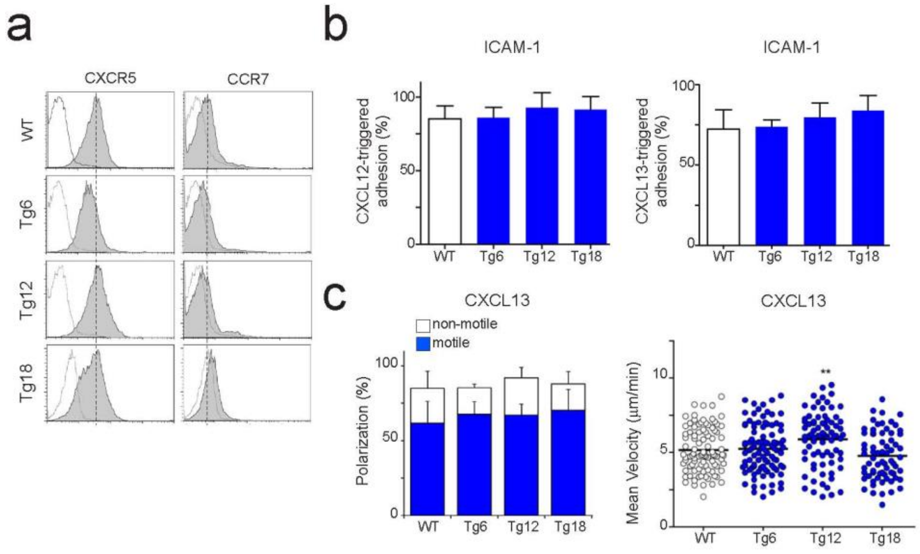

Supplementary Figure 6: Characterization of cell dynamics in NKX2-3 transgenic B cells. (a) Representative flow cytometry profiles (grey filled histogram) of the indicated molecules at the surface of WT B cells and NKX2-3 transgenic (Tg) B cells of distinct ages (Tg6, Tg12, and Tg18 are 6, 12, and 18-month-old mice, respectively). Dashed black line, maximum expression level in WT B cells. Dotted histogram, isotype control. (b) Adhesion frequencies to ICAM-1 membranes of WT and Tg-B cells in presence of CXCL12 (left) or CXCL13 (right) coating; data are the mean ± SD (*n* = 2). (c) Cell polarization frequencies, indicating the fractions of non-motile and motile cells, of WT and Tg-B cells settled on ICAM-1-membranes with CXCL13 coating (left); data are the mean ± SD (*n* = 2). Mean velocity values of motile B cells are shown (right); data are derived from two experiments and each dot is a single cell.

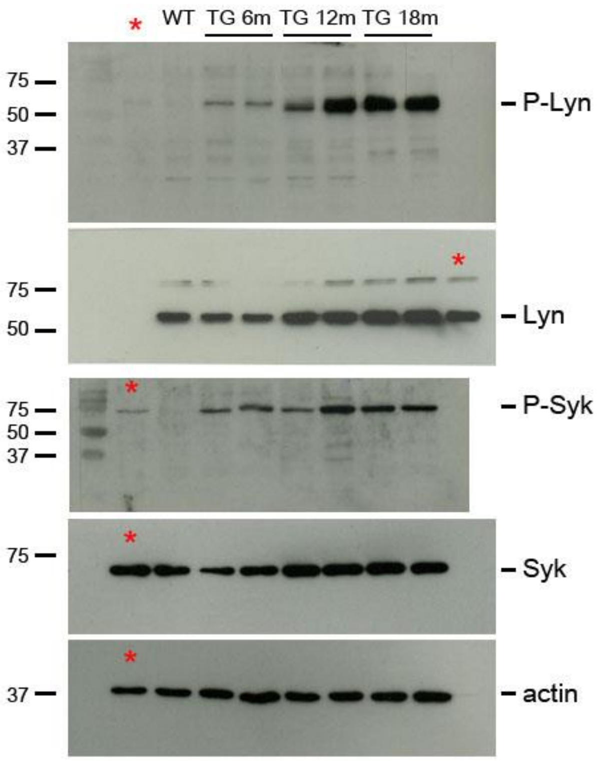

Supplementary Figure 7: Expression of BCR signaling in *NKX2-3* transgenic B cells. Uncropped version of the Western-blots shown in Figure 4e. Molecular weight markers (in kDa) are indicated (left). Red asterisk indicates internal control loaded in the Western-blot

SUPPLEMENTARY TABLES

Supplementary Table 1: Summary of the single nucleotide polymorphism identified by direct sequencing in the two coding regions of the NKX2-3 gene in human lymphoma cell lines and SMZL patient samples.

SNP	Reference	Consensus	Samples
rs41290504	C	A	Namalwa, Rec1, RCK8, SSK41<br>SMZL patient samples (4/19)
		M	OZ, SUDHL6, SUDHL8, HT<br>SMZL patient samples (9/19)
rs10529697	-	Largedeletion	SMZL patient samples (19/19)

Supplementary Table 2: FACS analysis of hematopoietic cell subpopulations in different tissues from WT and NKX2-3$^{-/-}$ deficient mice. The values represent the mean ± standard deviation (SD) of at least three mice.

Age (months)	4		8	
	WT	Nkx2-3$^{-/-}$	WT	Nkx2-3$^{-/-}$
**PERIPHERAL BLOOD**				
CD4	48.87 ± 15.01	35.17 ± 11.17	60.60 ± 4.16	37.35 ± 1.34<br>(P = 0.001)
CD8	17.93 ± 4.80	16.97 ± 4.92	18.50 ± 1.15	21.75 ± 3.75
B cells	28.23 ± 12.47	19.65 ± 6.58	27.93 ± 8.73	21.65 ± 3.32
**BONE MARROW**				
HSC	0.31 ± 0.07	0.28 ± 0.07	0.25 ± 0.09	0.19 ± 0.10
Pro B cells	1.04 ± 0.33	1.15 ± 0.04	0.41 ± 0.02	0.61 ± 0.19
Pre B cells	15.10 ± 1.87	13.70 ± 2.36	5.02 ± 0.94	4.84 ± 0.65
Immature B cells	5.88 ± 0.75	5.92 ± 1.70	3.46 ± 0.69	5.34 ± 0.16
Recirculating B cells	1.94 ± 0.79	1.48 ± 0.65	6.03 ± 0.77	5.34 ± 2.6

Age (months)	4		8	
	WT	Nkx2-3$^{-/-}$	WT	Nkx2-3$^{-/-}$
**THYMUS**				
CD4	6.46 ± 0.66	7.46 ± 1.99	7.75 ± 1.74	10.66 ± 4.44
CD8	1.65 ± 0.32	2.01 ± 0.27	1.49 ± 0.51	1.40 ± 0.40
CD4 CD8 DP	86.67 ± 1.26	83.93 ± 0.95	63.53 ± 11.97	78.90 ± 4.53
CD4 CD8 DN	2.50 ± 0.21	2.68 ± 1.51	2.59 ± 0.61	2.05 ± 0.27
**SPLEEN**				
CD4	50.13 ± 1.38	68.60 ± 4.69	38.90 ± 7.63	64.25 ± 4.60
CD8	22.13 ± 1.16	24.20 ± 1.97	16.57 ± 2.90	28.15 ± 1.06
B-Cells	40.13 ± 4.99	12.23 ± 2.01	24.43 ± 8.39	13.65 ± 3.04
T1 B Cells	6.87 ± 1.24	6.26 ± 3.14	7.52 ± 1.41	8.83 ± 3.35
Inmat. B-Cells	19.03 ± 4.65	7.86 ± 2.69	16.83 ± 0.85	9.79 ± 3.45
FO B-Cells	81.43 ± 4.28	89.87 ± 4.06	74.73 ± 4.34	87.55 ± 3.61
MZ B-Cells	6.94 ± 1.24	0.53 ± 0.22 (P = 0.0009)	12.57 ± 3.75	0.60 ± 0.06 (P = 0.005)
**LYMPH NODE**				
CD4	66.60 ± 7.24	60.43 ± 6.62	74.90 ± 1.70	60.90 ± 0.42
CD8	27.67 ± 1.50	32.87 ± 0.74	22.00 ± 2.83	36.20 ± 1.27
B cells	31.71 ± 3.54	28.87 ± 3.04	21.35 ± 8.70	21.25 ± 0.78

Supplementary Table 3: Peripheral blood counts obtained from Hemavet cell counter in WT and Eμ-*NKX2-3* transgenic mice. Data represent the mean ± standard deviation (SD) of at least three mice.

Age (months)	4			12			18		
Mice	WT	L1	L2	WT	L1	L2	WT	L1	L2
WBC (K/μL)	9.19 ± 2.65	4.86 ± 1.74 (P = 0.03)	8.53 ± 4.00	12.18 ± 2.51	6.20 ± 2.60 (P = 0.002)	6.43 ± 2.61 (P = 0.01)	10.53 ± 1.77	5.05 ± 2.44 (P < 0.0001)	6.23 ± 1.35 (P = 0.001)
NE (K/μL)	1.96 ± 0.69	1.35 ± 0.55	1.27 ± 0.19	2.61 ± 0.86	1.77 ± 0.67	1.63 ± 0.75	2.29 ± 0.67	2.05 ± 1.00	1.66 ± 0.86
LY (K/μL)	6.27 ± 1.28	3.03 ± 1.13 (P = 0.02)	5.08 ± 2.74	8.79 ± 1.54	4.01 ± 1.88 (P = 0.0005)	4.06 ± 1.61 (P = 0.004)	7.58 ± 1.43	2.55 ± 1.35 (P = 0.0004)	3.75 ± 1.00 (P = 0.006)
MO (K/μL)	0.75 ± 0.04	0.46 ± 0.21	0.56 ± 0.24	0.69 ± 0.15	0.28 ± 0.10 (P = 0.003)	0.52 ± 0.24	0.64 ± 0.21	0.33 ± 0.18 (P = 0.03)	0.43 ± 0.11
EO (K/μL)	0.18 ± 0.07	0.01 ± 0.01	0.08 ± 0.05	0.07 ± 0.10	0.11 ± 0.08	0.16 ± 0.09	0.01 ± 0.02	0.08 ± 0.05	0.11 ± 0.09
BA (K/μL)	0.02 ± 0.02	0.01 ± 0.01	0.01 ± 0.001	0.02 ± 0.02	0.03 ± 0.03	0.04 ± 0.03	0.01 ± 0.01	0.02 ± 0.02	0.005 ± 0.004
RBC (M/μL)	9.14 ± 0.21	9.58 ± 1.39	7.50 ± 1.05	9.58 ± 0.61	9.78 ± 1.81	8.82 ± 0.60	9.38 ± 1.08	9.19 ± 1.54	9.41 ± 1.13
Hb (gr/dL)	14.30 ± 0.41	15.27 ± 2.15	12.72 ± 0.75	14.52 ± 0.96	14.48 ± 2.42	14.85 ± 0.93	13.85 ± 1.15	14.39 ± 1.86	15.00 ± 1.63

Supplemental Table 4: Sequential FACS analysis of hematopoietic cell subpopulations (thymus, spleen, peripheral blood, bone marrow and lymph node) in WT and Eμ-NKX2-3 transgenic mice (L1 and L2) at 4, 12 and 18 months. Data represent the mean ± standard deviation (SD) of at least three mice.

Age (months)		4			12			18	
	WT	L1	L2	WT	L1	L2	WT	L1	L2
**THYMUS**									
CD4	3.19 ± 0.35	2.62 ± 0.58	3.05 ± 0.43	2.74 ± 0.97	2.57 ± 0.63	2.65 ± 1.32	2.99 ± 0.53	2.96 ± 0.73	2.82 ± 0.09
CD8	0.98 ± 0.10	1.05 ± 0.43	1.08 ± 0.32	1.16 ± 0.33	1.17 ± 0.49	1.15 ± 0.54	1.47 ± 0.64	1.18 ± 0.88	1.21 ± 0.15
CD4 CD8 DN	1.39 ± 0.22	1.70 ± 0.38	1.30 ± 0.18	1.41 ± 0.45	2.17 ± 1.02	1.99 ± 0.04	1.62 ± 0.75	1.87 ± 0.48	2.24 ± 0.44
CD4 CD8 DP	92.53 ± 0.12	92.87 ± 1.17	91.35 ± 1.21	92.60 ± 0.46	92.03 ± 0.49	89.65 ± 1.87	91.67 ± 1.70	86.60 ± 1.83	80.11 ± 1.27
**SPLEEN**									
CD4	53.07 ± 6.22	48.57 ± 6.00	49.03 ± 2.26	47.95 ± 6.02	49.98 ± 7.91	52.60 ± 3.81	51.88 ± 5.22	56.58 ± 9.60	52.65 ± 7.85
CD8	29.60 ± 1.68	27.67 ± 2.11	33.75 ± 2.47	28.12 ± 9.35	22.48 ± 6.99	27.25 ± 3.49	29.88 ± 3.50	18.48 ± 3.47 (P = 0.01)	31.05 ± 0.21
B-Cells	51.98 ± 3.29	39.92 ± 8.71	49.70 ± 0.74	54.80 ± 7.60	49.83 ± 8.83	54.85 ± 6.72	54.04 ± 4.13	51.43 ± 11.24	60.50 ± 0.42
T1 B Cells	17.53 ± 1.00	17.47 ± 4.35	18.43 ± 0.99	23.50 ± 2.21	18.72 ± 5.02	19.9 ± 1.13	21.82 ± 3.49	12.58 ± 0.85 (P = 0.03)	19.60 ± 0.75
Inmat. B-Cells	16.70 ± 3.47	15.62 ± 1.00	15.15 ± 0.49	13.60 ± 2.00	14.96 ± 6.77	12.9 ± 2.55	12.22 ± 2.80	10.65 ± 3.46	12.65 ± 0.07
FO B-Cells	78.65 ± 3.18	75.24 ± 3.33	83.80 ± 3.54	83.30 ± 1.04	73.82 ± 5.59 (P = 0.01)	79.28 ± 2.35	82.62 ± 4.95	67.05 ± 6.64 (P = 0.02)	82.58 ± 6.64
MZ B-Cells	5.37 ± 1.27	8.36 ± 1.79 (P = 0.02)	5.96 ± 1.85	5.67 ± 1.48	5.95 ± 1.46	4.99 ± 1.69	5.29 ± 1.57	6.11 ± 1.28 (P = 0.03)	5.10 ± 0.12

**LYMPH NODE**									
CD4	50.73 ± 12.45	59.40 ± 0.36	49.90 ± 5.09	53.27 ± 5.20	48.07 ± 6.96	48.22 ± 0.57	53.87 ± 1.81	52.47 ± 2.02	52.30 ± 0.71
CD8	33.13 ± 10.03	35.23 ± 0.99	42.30 ± 5.34	41.93 ± 8.34	48.40 ± 7.54	43.75 ± 1.24	42.77 ± 0.80	38.83 ± 0.95	40.95 ± 0.69
B-Cells	94.33 ± 0.81	92.93 ± 1.29	90.45 ± 1.23	93.07 ± 1.72	88.27 ± 4.48	90.64 ± 2,15	95.20 ± 0.98	87.57 ± 0.91	93.87 ± 2.13
**PERIPHERAL BLOOD**									
CD4	47.50 ± 1.91	43.30 ± 3.56	33.18 ± 1.76	29.18 ± 9.12	25.42 ± 10.79	21.53 ± 10.52	23.93 ± 3.50	10.57 ± 5.27 (P = 0.005)	15.95 ± 3.93 (P = 0.008)
CD8	32.53 ± 1.55	32.72 ± 2.27	37.05 ± 11.91	35.66 ± 9.14	33.60 ± 11.30	27.37 ± 12.88	34.82 ± 10.27	16.09 ± 9.28 (P = 0.01)	22.52 ± 4.54 (P = 0.005)
B-Cells	35.17 ± 2.94	24.08 ± 4.09 (P = 0.008)	34.07 ± 7.93	38.82 ± 5.26	31.23 ± 10.03	33.40 ± 10.27	51.47 ± 5.55	15.71 ± 7.22 (P = 0.02)	29.49 ± 7.45 (P = 0.01)
Granulocytes	26.23 ± 0.92	22.86 ± 7.76	24.05 ± 1.69	32.46 ± 6.80	28.58 ± 9.21	25.18 ± 3.25	40.73 ± 3.62	43.17 ± 7.52	41.98 ± 8.63
Macrophages	2.72 ± 0.59	2.37 ± 0.90	2.76 ± 1.88	2.19 ± 0.66	1.94 ± 0.47	2.59 ± 1.56	1.92 ± 0.68	1.89 ± 0.49	2.45 ± 1.04

Age (months)		4			12			18	
	WT	L1	L2	WT	L1	L2	WT	L1	L2
**BONE MARROW**									
pro & pre B-Cells	8.23 ± 1.31	7.52 ± 1.77	8.38 ± 0.23	3.94 ± 0.31	5.18 ± 0.96	5.89 ± 1.50	6.85 ± 1.53	7.42 ± 2.41	4.99 ± 0.10
pro B-Cells	1.23 ± 0.41	2.50 ± 1.47	1.16 ± 0.32	0.82 ± 0.38	1.73 ± 0.54	1.06 ± 0.65	0.94 ± 0.38	2.12 ± 0.93	1.10 ± 0.17
pre B-Cells	3.94 ± 1.03	2.49 ± 2.09	3.07 ± 1.78	0.98 ± 0.36	0.65 ± 0.43	0.85 ± 0.69	2.08 ± 1.03	1.44 ± 0.93	1.82 ± 0.62
Inmat. B-Cells	1.64 ± 0.38	1.32 ± 0.33	2.96 ± 0.09	0.78 ± 0.19	0.48 ± 0.20	1.53 ± 0.71	1.73 ± 0.21	0.98 ± 0.48	2.50 ± 0.13
Recirc. B-Cells	5.25 ± 0.50	5.25 ± 1.57	6.91 ± 0.48	5.60 ± 1.57	5.19 ± 2.45	4.45 ± 1.32	7.53 ± 2.23	3.31 ± 2.84 (P = 0.03)	10.1 ± 1.90
Granulocytes	54.50 ± 8.30	56.07 ± 7.64	49.40 ± 5.89	65.47 ± 5.99	54.40 ± 10.04	48.95 ± 0.21	60.70 ± 5.01	54.07 ± 1.86	58.40 ± 1.84
Macrophages	6.13 ± 1.18	5.90 ± 1.46	9.93 ± 2.36	8.31 ± 1.46	7.23 ± 0.85	7.23 ± 0.59	6.85 ± 1.76	8.81 ± 3.92	7.86 ± 1.87
HSC	0.76 ± 0.48	1.23 ± 0.79	0.70 ± 0.01	1.18 ± 0.30	1.05 ± 0.15	1.27 ± 0.40	0.95 ± 0.18	1.14 ± 0.21	0.66 ± 0.14

Supplementary Table 5: Anatomic location of lymphomas developed in Eμ-*NKX2-3* mice (L1 and L2).

ID	Line	Splenomegaly	Small intestine	Liver	Lung	Kidney	Salivary gland	Age (months)
6	1	YES	YES	NO	NO	YES	NO	20
39	1	YES	YES	YES	YES	NO	NO	23
785	1	YES	YES	NO	NO	YES	YES	19
782	1	YES	YES	YES	YES	YES	YES	18
757	1	YES	YES	NO	NO	YES	NO	19
761	1	YES	NO	NO	NO	NO	NO	19
202	1	YES	YES	YES	YES	YES	YES	20
846	1	YES	NO	NO	NO	NO	NO	18
962	1	YES	YES	NO	NO	YES	YES	19
964	1	YES	YES	NO	NO	YES	YES	20
956	1	YES	NO	NO	NO	NO	NO	17
168	1	YES	YES	NO	NO	YES	NO	18
484	1	YES	YES	NO	NO	NO	NO	23
498	1	YES	YES	YES	NO	YES	NO	23
489	1	YES	YES	YES	NO	YES	YES	16
497	1	YES	YES	YES	YES	YES	YES	12
669	1	YES	YES	YES	NO	YES	NO	17
627	1	YES	YES	NO	NO	NO	NO	18
257	1	YES	YES	NO	NO	NO	NO	19
180	1	YES	YES	YES	NO	YES	NO	18
179	1	YES	NO	YES	NO	NO	NO	22
687	1	YES	YES	YES	NO	NO	NO	20
646	1	YES	YES	YES	NO	YES	NO	18
582	1	YES	NO	NO	NO	NO	NO	12
563	1	YES	YES	YES	YES	YES	NO	12
580	1	YES	YES	NO	NO	YES	NO	12
194	2	YES	YES	YES	NO	YES	NO	25
833	2	YES	YES	YES	YES	YES	NO	25

622	2	YES	YES	NO	NO	NO	NO	16
142	2	YES	YES	YES	NO	NO	NO	22
169	2	YES	YES	YES	NO	NO	NO	20
831	2	YES	YES	YES	NO	YES	NO	20
826	2	YES	NO	NO	NO	YES	NO	20
581	2	YES	YES	YES	NO	YES	NO	18
487	2	YES	YES	NO	NO	NO	NO	24
833	2	YES	YES	YES	NO	YES	NO	25
841	2	YES	NO	NO	NO	NO	NO	25
999	2	YES	NO	NO	NO	NO	NO	18

Supplementary Table 6: High-resolution comparative genomic hybridization (aCGH) analysis of 14 clonal lymphomas developed in 18-month-old Eμ-NKX2-3 transgenic mice.

Number	ID	Gains	Losses
1	487	15qA1-qF3	11qE2
			16qC1.3-qC4
2	437	14qA1-qC3	8qD3
		15qA1-qF3	14qC3-qD3
		17qA1-qE5	16qC3.3-qC4
3	498	17qA1-qE5	6qC1
		15qA1-qF3	12qF1-qF2
			16qC3.3-qC4
			18qD1-qD3
4	445	17qA1-qE5	12qF1
			16qC3.3-qC4
5	179	14qD2	12qF1
		15qA1-qF3	17qE1.2
6	549	14qD3-qE5	17qE1.2
		10qB5.3	18qA2
7	687	2qA1-qH4	17qE1.2
		5qG1.1-qG3	
		10qA1-qD3	
		10qB5.3	
		11qB4	
		13qA1-qD2.3	
		14qA1-qE5	
		15qA1-qF3	
		17qA1-qE5	

8	6683	9qA1-qF4	2qC1.3-qE1
		10qA4	10qA1-qA2
		14qD2	10qA4-qB2
		14qD3-qE5	10qD1
		15qA1-qF3	11qA1-qA3.2
		18qA1-qE4	19qC1
		19qB-qD3	
		19qC2	
		19qD2	
9	400	2qA1-qB	2qB-qC1.3
		18qA1-qE4	2qC1.3-qE1
		5qF-qG3	2qE1-qH4
		9qA1-qA4	4qA1-qE2
		10qC2-qD1	5qA1-qF
		10qD2-qD3	8qA1.1-qE2
		11qE2	10qA1-qB5.3
		14qD2	10qA1-qA2
		14qD3-qE5	11qA1-qA3.2
		15qA1-qF3	11qA1-qC
		18qA1-qE4	12qA1.1-qF1
		19qC2	13qA3.1-qD2.3
			16qA1-qC4

Supplementary Table 7: List of the primers used in the study for gene sequencing, genotyping, gene expression (quantitative RT-PCR), and Q-PCR-ChIP analyses. All primers are listed as 5’-3’.

For sequencing

NAME	FORWARD	REVERSE
NKX2-3 exon1	GTCTGTCAAAAGCCCGACTC	CACCTCGTCCTTGTCTCTCC
NKX2-3 exon2	GCCATTTACTACCGCACG	CCCTGAGGAGCTAGACGTAC

For genotyping

NAME	SEQUENCE
12652cop	CCTGACCCTGCTTGCTCAACTCTACG
12563cop	TCATGTTCCTTGGGCTCGCTGC
12660PRO-JMC1	GACTTTTGCAGGCTCCACCAGACC
12661PRO-JMC1	AGCAAGCAGGGTCAGGCAAAGC

For gene expression

NAME	FORWARD	REVERSE
Human NKX2-3	GCTATGTCCACACGGTCCTG	CAGTCTCCGGCCGTCTCT
Mouse Nkx2-3	GCTTACAGCGGCAGCTA	GGTTGCTCACGTTCACAA
Human GAPDH	ACTTTGTCAAGCTCATTTCC	CACAGGGTACTTTATTGATG
Mouse Gapdh	ACTTTGTCAAGCTCATTTCC	TGCAGCGAACTTTATTGATG

For pyrosequencing analysis of NKX2-3

PRIMERS	SEQUENCE	PCR product
NKX2-3-D	GTGACGTACTAGCAACGGTTTTTGTAGTGGTTGTA	232 bp
NKX2-3-R	TAGCAGGATACGACTATCAAAACCACTTAATTATCCAATCCAA	
USF	GTGACGTACTAGCAACG	
BIOTIN-USR	(bio)TAGCAGGATACGACTATC	

INTERMEDIATE PYROSEQUENCING PRIMERS	
NKX2-3-PYR-1<br>NKX2-3-PYR-2<br>NKX2-3-PYR-3	GTTTTTGTAGTGGTTGTAATAAAATTTAGA<br>GATTTAGATTGGAGTGGGA<br>GGAGTTTAGGAGGAGAGTTGGA

For Q-PCR-ChIP analysis

NKX2-3	PRIMERS/ PROBES	SEQUENCES	PCR product
Promoter	NKX2-3-P-D<br>NKX2-3-P-R<br>NKX2-3-P-P	CAGGCAGGCACATACAGCTA<br>CCTGCAGCTTGTGTTAGCAA<br>FAMGGGAAGTGATAAGTGACATGCATAMRA	79 bp
First exon	NKX2-3-E-D<br>NKX2-3-E-R<br>NKX2-3-E-P	GTCCCTGCAGTGGCTGTAAC<br>CGGTCCCACTCCAGTCTAAA<br>FAMAAACCCAGACCCCCAGGTTAMRA	77 bp

For LDI-PCR studies

PRIMER	SEQUENCE	APPLICATION
J6E	CCCACAGGCAGTAGCAGAAAACAA	External J6 primer
J6I	TCTGGGCTCGAGTCGACGCAGAAAACAAAGGCCCTAG AGGG	Internal J6 primer
JBE	GAAGCAGGTCACCGCGAGAGT	External primer for BglII digests
JBI	CTTCTGGTTGTGAAGAGGTGGTTTTG	Internal primer for BglII digests
JHE	TGGGATGCGTGGCTTCTGCT	External primer for HindIII digests
JHI	GCCCTTGTTAATGGACTTGGAGGA	Internal primer for HindIII digests
JXE	CACTGGCATCGCCCTTTGTCTAA	External primer for XbaI/PstI digests
JXI	CCCATGCCTTCCAAAGCGATT	External primer for XbaI/PstI digests

Supplementary Table 8: Antibodies used in the study for immunohistochemistry (IHC), immunofluorescence (IF), Western blot (WB) and EMSA (h, human; m, mouse).

ANTIBODY	FROM	USE
Nkx2-3 454C (h, m)	cf. Methods	IHC, IF (1:200)
Actin (h, m)	Oncogene Research, Merck	WB (1:1,000)
CD20 (m)	Dako Cytomation	IHC (1:50)
CD3 (m)	Santa Cruz	IHC (1:20)
IgM (m)	Serotec	IHC (1:50)
IgD (m)	Monosan	IHC (1:50)
CD10 (m)	Santa Cruz	IHC (1:50)
TdT (m)	Abcam	IHC (1:100)
Gcet1 (m)	Abcam	IHC (1:100)
Mum1 (m)	Abcam	IHC (1:100)
Bcl2 (m)	Epitomics	IHC (1:200)
Bcl6 (m)	Santa Cruz	IHC (1:100)
Bcl10 (m)	Santa Cruz	IHC (1:100)
Foxp1 (m)	Abcam	IHC (1:200)
MadCAM-1 (m)	Santa Cruz	IF
IBL-11 (m)	Ref. 60	IF
IgM (m)	Serotec	IF
MOMA-1 (m)	AbD Serotec	IF
MARCO (m) clone IBL-12	Ref. 59	IF
CD21/35 (m)	BD Biosciences	IF
VCAM-1 (m)	BD Biosciences	IF
ICAM-1 (m)	Provided by Dr. Szakal	IF
IBL-7/1 (m)	Ref. 61	IF
IBL-9/2 (m)	Ref. 61	IF
Sn/CD169 (m) clone IBL-13	Ref. 59	IF
CR1/2/CD21 (m) clone 7G6	BD Biosciences	IF
B220 (m) clone RA3-6B2	BD Biosciences	IF
NF-kB2 p100/p52 (m)	Cell Signaling	WB (1:1,000)
IkB-α (m)	Cell Signaling	WB (1:1,000)
Phospho-IkB-α (m)	Cell Signaling	WB (1:1,000)
NF-kB1 p65 (m)	Cell Signaling	WB (1:1,000)
Phospho-NF-kB1 p65 (m)	Cell Signaling	WB (1:1,000)
SAPK/JNK (m)	Cell Signaling	WB (1:1,000)
Phospho-SAPK/JNK (m)	Cell Signaling	WB (1:1,000)
p38 MAPK (m)	Cell Signaling	WB (1:1,000)
Phospho p38-MAPK (m)	Cell Signaling	WB (1:1,000)
p105/p50 (h, m)	Abcam	WB (1:1,000)
c-Rel (h, m)	Cell Signaling	WB (1:1,000)
RelB (h, m)	Cell Signaling	WB (1:1,000)
Cdk4 (h, m)	BD Biosciences	WB (1:1,000)
Cdk6 (h, m)	Abcam	WB (1:1,000)
Cyclin D2 (h, m)	Abcam	WB (1:1,000)
Cyclin D3 (h, m)	Abcam	WB (1:1,000)
Phospho-AKT (m, h)	Abcam	WB (1:1,000)

AKT (m, h)	Abcam	WB (1:1,000)
Phospho-Y416 Src (detects phosphor-Y397 Lyn) (h, m)	Cell Signaling	WB (1:1,000)
Lyn (m, h)	Cell Signaling	WB (1:1,000)
Phospho-Y352 Syk (h, m)	Cell Signaling	WB (1:1,000)
Syk (h, m)	Cell Signaling	WB (1:1,000)
NFkB p65 (C-20)	Santa Cruz	EMSA (1:1,000)
NFkB p50 (4D1)	Santa Cruz	EMSA (1:1,000)
c-Rel (N)	Santa Cruz	EMSA (1:1,000)
RelB (C-19)	Santa Cruz	EMSA (1:1,000)
NFkB p52 (447)	Santa Cruz	EMSA (1:1,000)

Supplementary Table 9: Antibodies used for flow cytometry.

**Lineage Marker**	**Clone**	**Lineage Marker**	**Clone**
CD3e	145-2C11	Ly-6G (Gr1)	RB6-8C5
CD4	RM4-5	CD11a	2D7
CD8	53-6.7	CD49d	R1-2
CD11b (Mac-1)	M1/70	CD44	IM7
CD19	1D3	CD54	3E2
CD16/CD32 (Fc Block)	2.4G2	CD106	429
CD21	7G6	CD62L	MEL-14
CD23	B3B4	CD80	
CD25	PC61	CD86	
CD45R/B220	RA3-6B2	CD95 (FAS)	2G8
CD117 (cKit)	2B8		
IgD	11-26c.2a		
IgM	R6-60.2		
Ly-6A/E (Sca1)	E13-161.7		
